# WISP-1/CCN4 Regulates Osteogenesis by Enhancing BMP-2 Activity

**DOI:** 10.1002/jbmr.205

**Published:** 2010-08-03

**Authors:** Mitsuaki Ono, Colette A Inkson, Tina M Kilts, Marian F Young

**Affiliations:** Craniofacial and Skeletal Diseases Branch, National Institutes of Craniofacial and Dental Research, National Institutes of HealthBethesda, MD, USA

**Keywords:** WISP1, GROWTH FACTORS, BMP, CYTOKINES, OSTEOPROGENITORS, CCN

## Abstract

Wnt-induced secreted protein 1 (WISP-1/CCN4) is a member of the CCN family that is highly expressed in skeletal tissue and in osteoprogenitor cells induced to differentiate in vitro. To determine the function of WISP-1 during osteogeneis, osteogenic bone marrow stromal cells (BMSCs) were transduced with WISP-1 adenovirus (adWISP-1) in the presence or absence of bone morphogenetic protein 2 (BMP-2) adenovirus (adBMP-2). WISP-1 overexpression enhanced the ability of BMP-2 to direct BMSCs toward osteogenic differentiation and appeared to work by stimulating Smad-1/5/8 phosphorylation and activation. The ability of WISP-1 to enhance BMP-2 activity also was shown in vivo using an ectopic osteogenesis assay with BMSCs transduced with WISP-1, BMP-2, or both. When BMSCs were infected with lentivirus containing human *WISP1* shRNA, they formed less bone in vivo and were less responsive to BMP-2, confirming that WISP-1 and BMP-2 have a functional interaction. Immunoprecipitation (IP) and Western blot analysis showed that WISP-1 bound directly to BMP-2 and showed that WISP-1 increased BMP-2 binding to hBMSCs in a dose-dependent fashion. To understand how WISP-1 enhanced BMP-2 signaling, the influence of WISP-1 on integrin expression was analyzed. WISP-1 induced the mRNA and protein levels of α_5_ -integrin and, further, was found to bind to it. Antibody-blocking experiments showed that the BMP-2 binding to BMSCs that was enhanced by WISP-1 was completely neutralized by treatment with anti-integrin α_5_ β_1_ antibody. Pilot studies and the use of transgenic mice that overexpressed human WISP-1 in preosteoblasts had increased bone mineral density (BMD), trabecular thickness, and bone volume (BV/TV) over wild-type controls, supporting observations using human osteoprogenitors that WISP-1 has a positive influence on osteogenesis in vivo. In conclusion, these studies show, for the first time, that WISP-1 has a positive influence on bone cell differentiation and function and may work by enhancing the effects of BMP-2 to increase osteogenesis through a mechanism potentially involving binding to integrin α_5_ β_1_ . © 2011 American Society for Bone and Mineral Research.

## Introduction

With an aging world population, the search for more efficient drug targets for age-related disorders such as osteoporosis and osteoarthritis has become essential. The use of antiresorptive drugs has prevailed as the most common treatment of osteoporosis. However, in more recent years, the use of anabolic agents such as parathyroid hormone (PTH) and bone morphogenetic protein 2 (BMP-2) has become more common. Most recently, the search for biologic proteins that can increase bone mass has focused on the Wnt signaling pathway owing to reports of high bone mass in people with mutations in the Wnt receptor LRP5/6. Wnt-induced secreted protein 1 (WISP-1/CCN4) is a member of the CCN family that is highly expressed in skeletal tissues. Evidence points to a role for WISP-1 in bone formation and maintenance. Expression of WISP-1 has been observed in the developing skeleton and later in both preosteoblastic and osteoblastic cells,(1) specifically at sites of new bone formation during development or in healing fracture calluses.(2) *WISP1* was identified initially as the principle target gene of the Wnt-1/Frizzled pathway in breast cancer cells, with its expression regulated by β-catenin.(3) The Wnt/β-catenin signaling pathway has proved to be essential in the regulation and maintenance of bone mass,(4,5) and mutations in *LRP5/6* that prevent binding to its inhibitor, dickkoff 1, cause high-bone-mass phenotype in humans.(6–8) Moreover, *WISP1* has been identified as a gene that is highly upregulated in osteoarthritis.(9,10) Previous work from our lab has shown that recombinant WISP-1 promotes the proliferation and differentiation of human bone marrow stromal cells (hBMSCs) in vitro via a relationship with members of the transforming growth factor β (TGF-β) superfamily.(11) However, the exact skeletal functions of WISP-1 in vivo or the molecular mechanism involved have not yet been elucidated.

The CCN family consists of its three founding members, Cyr61 (CCN1/Cyr61/Cef10), connective tissue growth factor/CTGF (CCN2/CTGF/Fisp12), and Nov (CCN3/Nov)(12,13) and the later identified WISP-1 (CCN4/rCOP-1/Elm-1), WISP-2 (CCN5), and WISP-3 (CCN6). CCN family proteins are characterized by four distinct functional domains: (1) an insulin-like growth factor–binding protein–like module (IGFBP), (2) a von Willebrand factor type C repeat (VWC), (3) a thrombospondin type 1 repeat (TSP1), and (4) a cysteine-rich C-terminal module (CT). CCN5 is the exception, being a trimodular protein lacking the CT module. Numerous studies have demonstrated a role for the CCN family members in the regulation the osteo/chondrogenic differentiation of musculoskeletal cells both in vitro and in vivo.(12,14–19) In addition, the CCN family has been implicated in the pathology of a number of fibrotic disorders(20) and several types of malignancies, including breast,(21,22) prostate,(23–25) and gastric cancers,(26,27) and have functions identified in numerous biologic processes such as embryonic development, angiogenesis, cell differentiation, and tissue repair (for a recent review see refs. (2), (12), (17), (28), and (29)). At the cellular level, CCN proteins can act as growth factors to stimulate differentiation and collagen synthesis(13,30) or to mediate cell binding, migration, and signaling via interactions with integrins.(31–38) In addition, CCN family members also have been identified as factors that can regulate members of the TGF-β superfamily.(39–41)

Bone morphogenetic proteins (BMPs) are members of the TGF-β superfamily of polypeptides.(42,43) A fundamental function of BMPs is to induce the differentiation of mesenchymal osteoprogenitors toward cells of the osteoblastic lineage and then to promote osteoblastic maturation and function. This cellular regulation, in turn, can control the development of bone and cartilage and the accelerated healing of fractured bones. While BMPs play essential roles in bone formation and remodeling, there is still a need to modulate their activities.(42,43) Numerous antagonists of BMPs exist, such as noggin, chordin, short gastrulation (Sog), and twisted gastrulation (Tsg),(42,43) that control BMP activity. The modulation of BMP activity also can be achieved by local feedback mechanisms, involving growth factor–binding proteins and other intracellular factors. Indeed, the CCN proteins CCN2/CTGF and CCN3/Nov both bind to BMPs and temper their functions in vitro and in vivo.(15,16) These studies suggest that the CCN family is involved in regulation of osteoblast differentiation by interacting with members of the TGF-β family such as BMP-2, a growth factor long known to be an important modulator of skeletogenesis.(44) We have shown previously a cooperative relationship between WISP-1 and TGF-β in regulating osteoblastic proliferation and differentiation. However, the relationship of WISP-1 to BMP-2 in bone formation has not yet been determined.

To deepen our understanding of the function of WISP-1 in osteogenesis, we investigated the effect of WISP-1 on BMP-2-induced osteogenic differentiation and found that WISP-1 potentiates the effects of BMP-2 in progenitor cells from the bone marrow. The importance of WISP-1 in enhancing BMP-2 function in vivo was determined using an ectopic osteogenesis assay. Evidence points to an essential role of CCN family members in integrin binding and signaling; therefore, we also determined the effects of WISP-1 on integrin production and function and found a functional relationship between WISP-1 and α_5_ β_1_ integrin that can regulate BMP-2 bioavailability in hBSMC cultures. Taken together, these data present a new paradigm revealing that WISP-1 has enhancing effects on BMP function during osteogenesis that are potentially dependent on the integrin α_5_ β_1_ .

## Materials and Methods

### Cells and culture medium

Human BMSCs (hBMSCs) were isolated using methods reported previously.(11) All specimens were used in accordance with the NIH regulations governing the use of human subjects under Institutional Review Board (IRB) exemption (D-0188). hBMSC cultures were maintained in α-minimal essential medium (α-MEM, Invitrogen, Carlsbad, CA, USA) containing 20% fetal bovine serum (FBS), 2 mM glutamine, 100 U/mL of penicillin, and 100 mg/mL of streptomycin sulfate (Invitrogen). In all experiments, hBMSCs were used between the third and seventh passage. To induce cells toward an osteoblast-like phenotype, cultures were supplemented with 100 mg/mL of l-ascorbic acid (Wako, TX, USA), 1.8 mM of KH_2_ PO_4_, and 10 nM of dexamethasone (Sigma, St Louis, MO, USA). Mouse BMSCs (mBMSCs) were isolated as described previously(45) and cultured in α-MEM containing 20% lot-selected FBS, 2 mM of glutamine, 100 U/mL of penicillin, 100 mg/mL of streptomycin sulfate, 10 nM of dexamethasone, and 55 µM of 2-mercaptoethanol (Invitrogen). The osteogenesis-inducing medium was supplemented with 100 mM of ascorbic acid 2-phosphate and 2 mM of β-glycerophosphate (Sigma).(11)

C2C12 mouse myoblast cells, which have a stably integrated reporter plasmid consisting of the BMP-responsive element from the Id promoter fused to a luciferase reporter,(46) were kindly supplied by Drs L Zilberberg and D Rifkin (New York University School of Medicine, New York, NY, USA). C2C12 cells were cultured in Dulbecco's Modified Eagle's Medium (DMEM) containing 10% FBS, 100 U/mL of penicillin, and 100 mg/mL of streptomycin sulfate.

### Generation and transduction of adenovirus

Adenoviruses encoding human WISP-1 were generated using the *BamHI*/*NotI* fragment of full-length human *WISP1* cDNA,(11,47) cloned into the adenovirus vector VQ ad5 DMV K-NpA (Viraquest, North Liberty, IA, USA). The resulting plasmid, named adWISP-1, was subject to homologous recombination, and the large-scale amplification of purified plaques was carried out by Viraquest. Adenovirus encoding murine BMP (adBMP-2)(48) was the kind gift of Dr R Francheschi (University of Michigan, Ann Arbor, MI, USA) and was amplified and purified by Viraquest. For transduction of adenovirus, the cells were first grown to 80% confluency and then incubated with medium containing an adenovirus (hBMSCs: 5 × 10^3^ particles/cell; C2C12 cells: 1 × 10^3^ particles/cell). Optimal viral doses for transduction of hBMSCs or C2C12 cells were determined by serial plaque-forming unit (PFU) dilution and tested by Western blotting (see Supplemental Fig. S1) or by real-time PCR (not shown). Cells were used 72 hours after transduction unless otherwise specified.

### Lentivirus production and transduction

Lentiviral constructs carrying shRNA targeting human *WISP1* mRNA were purchased from OpenBioSystems (Huntsville, AL, USA). The pLKO.1 gene set for *WISP1* contained five different clones: AXA20-B10, AAE91-A-7, AAE91-A-8, AAE-91-9, and AAE91-A10. The plasmid pLKO.1 eGFP lentivirus was used as a control (GFP). Plasmid DNA was amplified and purified using CsCl gradients and then transfected along with packaging plasmids into HEK293T cells to generate VSV-g pseudotyped lentivirus particles. The cells were refed with DMEM medium, 10% FBS, and 1% Penn-Strep for 24 hours after transfection, and the culture supernatant was harvested 48 hours after transfection. The supernatants containing infective lentivirus were clarified by centrifugation and filtration and used to infect hBMSC culture using the same culture procedures described earlier except that 2 µg/mL of puromycin was added to select cells with integrated DNA. After antibiotic selection for 2 days, cells were analyzed for relative production of *WISP1* mRNA using real-time PCR, and the clone with the greatest reduction in relative expression of *WISP1* was used for in vitro differentiation and in vivo osteogenesis assays.

### In vivo osteogenesis assay

For the in vivo osteogenic ectopic assay 3 × 10^6^ shRNA lentivirus- or GFP control virus-infected hBMSCs were mixed with 40 mg of hydroxyapatite/tricalcium phosphate powder (Warsaw, IN, USA) and implanted under the skin on the backs of 8-week-old athymic nude mice (Harlan, Madison, WI, USA). After 6 weeks, the transplants were harvested and fixed and embedded in paraffin, serial 6-µm sections were stained with hematoxylin and eosin, and quantification of de novo bone formation was performed using a BZ-8000 microscope equipped with a BZ analyzer (Keyence, Osaka, Japan). Specifically, three separate areas of the implant was traced and then tabulated using the BZ software, which determined the percent bone area/total area. The same procedure was used for the gain-of-function experiments except that hBMSCs were transduced with adenovirus as described earlier and harvested at 4 weeks instead of 6 weeks.

### Reverse-transcriptase polymerase chain reaction (RT-PCR) and real-time RT-PCR analysis

Total cellular RNA was extracted using RNeasy (Qiagen, Gaithersburg, MD, USA) according to the manufacturer's protocol and cDNA produced by reverse transcription of RNA using the iScript cDNA Synthesis Kit (Bio-Rad, Hercules, CA, USA). PCR amplification of cDNA was carried out using GoTaq Green Master Mix (Promega, Madison, WI, USA) employing gene-specific primer sets (listed in Table 1). Primers were designed using Beacon Software from Bio-Rad with parameters set for use with real-time PCR and for a *T*_*m*_ of 62°C ± 3°C with an amplicon size of 75 to 200 bp (Table 1). Real-time RT-PCR was performed to quantify the expression of mRNA using a MyiQ (Bio-Rad) with iQ SYBR Green Supermix (Bio-Rad). For each primer set, melting curves were performed to ensure that a single peak was produced, and the products were analyzed by gel electrophoresis. All real-time PCR reactions were normalized to the levels of *ribosomal protein S29* (*s29*) mRNA for real-time PCR or to the levels of *Gapdh* for semiquantitative RT-PCR.

**Table 1:** Primers Used for RT-PCR and Real Time RT-PCR Experiments

Gene	GenBank accession no.	Primer sequence	PCR product length (bp)
Gapdh^a^^,^^c^	BC082592	5′-GAGAGGCCCTATCCCAACTC-3′ (S)	148
		5′-GTGGGTGCAGCAGCGAACTTAT-3′ (AS)	
*Wisp-1*^a^^,^^c^	BC048791	5′-TGGCAGCAGTGACAGCAGCA-3′ (S)	355
		5′-GACCTGTGACCTTTAGGTGTGA-3′ (AS)	
*WISP-1*^b^^,^^d^	AF100779	5′-TGGCAGCAGTGACAGCAGCA-3′ (S)	609
		5′-TACGGAGGTGGAGTGGGTGT-3′ (AS)	
*S29*^b^^,^^d^	BC032813	5′-TCTCGCTCTTGTCGTGTCTGTTC-3′ (S)	75
		5′-ACACTGGCGGCACATATTGAGG-3′ (AS)	
*ALP*^b^^,^^d^	NM_000478	5′-GCACCGCCACCGCCTACC-3′ (S)	150
		5′-CCACAGATTTCCCAGCGTCCTTG-3′ (AS)	
*OPN*^b^^,^^d^	NM_000582	5′-CTGTGTTGGTGGAGGATGTCTGC-3′ (S)	143
		5′-GTCGGCGTTTGGCTGAGAAGG-3′ (AS)	
*WISP-1*^b^^,^^d^	AB034725	5′-ACACTCATTAAGGCAGGGAAGAAG-3′ (S)	185
		5′-TCAGGACACTGGAAGGACACG-3′ (AS)	
*Integrina5*^b^^,^^d^	NM_002205	5′-GGGCTGGATGACTTGCTGGTG-3′ (S)	125
		5′-GTGGGCGTGGGCTCTATGC-3′ (AS)	
*Integrinav*^b^^,^^d^	BC047454	5′-AGCGGGACCATCTCATCACTAAGC-3′ (S)	86
		5′-CAAGCACTGAGCAACTCCACAACC-3′ (AS)	
*Integrinb1*^b^^,^^d^	BC020057	5′-TGGGCTTTACGGAGGAAGTAGAGG-3′ (S)	90
		5′-GACACTTGGGACTTTCAGGGATGC-3′ (AS)	
*Integrinb3*^b^^,^^d^	NM_000212	5′-TAGAAGAGCCAGAGTGTCCCAAGG-3′ (S)	128
		5′-TCGGTCGTGGATGGTGATGAGG-3′ (AS)	

S, sense; AS, antisense.

a mouse

b human

c RT-PCR

d real time RT-PCR.

### In vitro calcium accumulation

Calcium deposits were detected by staining with 2% alizarin red S (pH 4.2, Sigma) and quantified by elution of bound alizarin red with 0.5 mL of 5% SDS in 0.5 N HCl for 30 minutes at room temperature and measuring the absorbance at 405 nm.

### Solid-phase binding assay

Polystyrene microtiter plates were coated with bait proteins prepared in a 0.1% BSA/PBS solution by overnight incubation at 4°C. Unbound protein was removed, and nonspecific binding sites were blocked with 1% BSA in PBS for 1 hour at room temperature. Bait-coated plates were incubated with test proteins prepared in 1% BSA/PBS solution for 2 hours at room temperature. Following washes with 1% BSA/PBS solution, protein binding was assayed by incubating with primary antibody to the proteins being tested for 1 hour, after which the wells were washed and incubated for 1 hour with goat antirabbit horseradish peroxidase (HRP) conjugate (Pierce, Thermo Scientific, Rockford, IL, USA). At the end of the incubation period, the wells were washed six times with PBS containing 0.05% Tween 20 before visualization of signal using an HRP chromogenic substrate (TMB, Kirkegaard & Perry Laboratories, Gaithersburg, MD, USA). The reaction was stopped with 1 M phosphoric acid, and the absorbance at 450 nm was measured. Nonspecific binding was determined in parallel incubations by omitting microtiter well coating.

### Immunoprecipitation

Cells were harvested using NP-40 buffer (1% NP-40, 0.15 M NaCl, 0.01 M sodium phosphate, and 1 mM EDTA) containing a Complete Protease Inhibitor Mixture (Roche Diagnostics, Mannheim, Germany). Cleared cell lysates were incubated with anti-BMP-2 (sc-6895, Santa Cruz Biotechnology, Santa Cruz, CA, USA) or anti-integrin α_5_ β_1_ (Millipore, Bellierica, MA, USA) antibodies that were bound to Dynabeads protein G (Dynal, Invitrogen). The beads were washed extensively, and bound proteins were boiled in loading buffer for 5 minutes and separated by SDS-PAGE and analyzed by immunoblot with anti-WISP-1 antibody (LF-187). Details on the construction and specificity of LF-187 have been described previously.(49)

### Western blot analysis

Total cellular proteins were prepared by lysing cells in M-PER mammalian protein extraction reagent (Pierce) or PhosphoSafe Extraction Reagent (Novagen, Darmstadt, Germany) supplemented with a protease inhibitor cocktail (1 µg/mL; Roche, Indianapolis, IN, USA). Following centrifugation at 10,000 rpm for 20 minutes at 4°C to remove cell debris, the protein content of the cell lysate was determined using a BCA assay kit (Pierce). Between 1 and 30 µg of total protein was separated by electrophoresis on 4% to 12% Bis-Tris precast polyacrylamide gels (NuPage, Invitrogen) using MOPS buffer (Invitrogen) and then transferred onto polyvinylidene fluoride (PVDF) membranes (Immobilom-FL, Millipore) at 100 V for 2 hours with cooling. Blots were blocked with Odyssey Blocking Buffer (Licor, Lincoln, NE, USA) for 1 hour at room temperature before overnight incubation at 4°C with primary antibodies to WISP-1 (LF-187 1:1000), pSmad-1/5/8 and total Smad-1 (1:1000; Cell Signaling Technologies, Danvers, MA, USA), and bCBFA1/AML3 (1:100; Calbiochem, La Jolla, CA, USA). Membranes were probed with fluorescently labeled secondary antibody (1:10,000; Licor), again diluted in blocking solution and incubated for 1 hour at room temperature. After washing with Tris-buffered saline with Tween (TBS-T), membranes were scanned with an Odyssey scanner (Licor). Blots were stripped of antibodies using OneMinute Western Blot Stripping Buffer (GM Biosciences, Inc., Frederick, MD, USA) and reprobed to determine relative loading efficiency using antibodies to HSP-90 (1:2000; Santa Cruz Biotechnology), or β-actin (1:10,000; Sigma).

### BMP-responsive luciferase reporter assay

Stably transfected C2C12 cells expressing the BMP-responsive mouse Id promoter were used to assay BMP activity. BRE-luc C2C12 cells (4 × 10^3^ cells/well) were plated into white clear-bottomed 96-well plates. After 24 hours, cells were infected with adWISP-1, adBMP-2, or both (multiplicity of infection [MOI]: 1000 PFU). Then 72 hours after infection, a Steady-Glo Reagent (Promega) was added in each well, and luciferase activity was measured using a microplate luminometer (Berthold, Wildbad, Germany).

### “On cell” binding

To analyze the effect of α_5_ β_1_ intergrin on WISP-1-induced BMP-2 binding, human BMSCs were seeded at 1 × 10^4^ cells/well in 96-well plates and preincubated with 10 mg/mL of anti-α_5_ β_1_ integrin antibody (Millipore) or IgG control for 1 hour and then transduced with adWISP-1. After 2 days, the cells were fixed for 60 minutes in 4% phosphate-buffered formaldehyde at room temperature, washed with PBS, and blocked in Odyssey Blocking Buffer (Licor) for 1 hour at room temperature. The affinity of BMP-2 to bind to hBMSCs in response to WISP-1 overexpression then was tested by incubating with BMP-2 (10 µg/mL; R&D, Minneapolis, MN, USA) for 2 hours at room temperature. Cells treated without BMP-2 were used to distinguish endogenous BMP-2 and served as negative controls. Bound BMP-2 was identified by incubation of anti-BMP-2 antibody (2.5 µg/mL; Santa Cruz Biotechnology) for 1 hour and fluorescently labeled secondary antibody (1:200; Licor) for 1 hour at room temperature. To detect levels of cell surface–associated WISP-1, cells were incubated with WISP-1 antibody (LF-187, 1:200) for 1 hour and then washed and incubated with fluorescently labeled secondary antibody (1:200; Licor) for 1 hour at room temperature. The relative levels of fluorescently bound secondary antibody were measured using an Odyssey scanner (Licor).

### Generation of transgenic mice overexpressing human *WISP1*

A plasmid containing a full-length human *WISP1* cDNA was the kind gift of Arnold Levine (Rockefeller University, New York, NY, USA). A 1.3-kb *WISP1* fragment was purified from pBabe-Puro retroviral DNA vector and subcloned into pcDNA3.1– (Invitrogen) using *BamHI* and *EcoRI* sites. Large-scale synthesis and purification of endotoxin-free plasmids were carried out using standard techniques and the Qiagen Endo-Free Maxi Preparation Purification Kit, and the integrity of the construct was confirmed by DNA sequencing. The Col1A1 promoter (−2310 to +110) was the kind gift of Benoit deCrombrugge (MD Anderson Cancer Center, Houston, TX, USA). Following release from the pJ251 using *Asp718* and *BamH1* sites, the promoter was cloned immediately upstream of *WISP1* in pcDNA3.1– to produce a final construct of 9.12 kb (*Col1A1-WISP1* pcDNA). Transgenic mice that express *WISP1* under control of the Col1A1 promoter were generated using a 4.8-kb DNA fragment including the Col1A1 promoter (2.3 kb of promoter, 0.1 kb of exon 1), WISP-1 (1.3 kb), and the bovine growth hormone polyA tail (BGH pA) (1.1 kb), which was excised from *Col1A1-WISP1* pcDNA using *ASP718* and *StuI* and purified by sucrose gradient. Transgenic mice were prepared by pronuclear injection with assistance from the NIDCR-DIR Functional Genomics Core Facility. Six founder mice were identified that contained the transgene, as judged by Southern blot analysis of DNA isolated from mouse tails using the 4.8-kb fragment generated for pronuclear injection as the probe. Two of the founder lines showed high levels of transgene integration. One mouse line that overexpressed the transgene, as detected by Western blot analysis of mouse bone, was identified and was studied in greater detail subsequently. A PCR strategy was devised for routine genotyping using primers in the 3' end of the col1A1 promoter (5'-TGGACTCCTTTCCCTTCCTT-3') and the 5' end of the human *WISP1* cDNA (5'-GCAGGAACCACCTCATGC-3').

### X-ray and micro–computed tomographic (µCT) analysis

Femurs were dissected from wild-type and *WISP1* transgenic mice and subject to radiography using Kodak X-OMAT TL film (Rochester, NY, USA) and a Model FX-20 Faxitron X-ray system (Lincolnshire, IL, USA) at a setting 30 kV using a 40-second exposure time. The femurs were scanned and reconstructed with 8-µm isotropic voxels on a µCT analysis system (eXplore MS, GE Medical Systems, London, Ontario, Canada). A bone standard (SB3, Gammex RMI, Middleton, WI, USA) was scanned with the µCT and used for the calibration of bone mass measurements. Reconstructed 3D images of distal femurs were analyzed using a Microviewer (GE Medical Systems). A fixed threshold was used to separate the bone and marrow phases. The trabecular bone mineral density (BMD), trabecular bone volume per tissue volume (BV/TV), bone surface per bone volume (BS/BV), trabecular thickness (Tb.Th), trabecular number (Tb.N), and trabecular spacing (Tb.Spac) in the distal femur were measured in a rectangular cylinder within the metaphysis. Cortical surface area (CSA), cortical area (CA), marrow area (MA), and cortical thickness (CT) were measured in a rectangular circle in the diaphysis.

### Statistics

One-way factorial analysis of variance (ANOVA) followed by Tukey tests was used for the statistical analysis (Prism 5, GraphPad Software, Inc., La Jolla, CA, USA). *p* values of less than .05 were considered to be statistically significant. All statistical data were presented as the mean ± SD.

## Results

### Expression of WISP-1 in human bone marrow cultures during osteoblastic differentiation

**Fig. 1:**
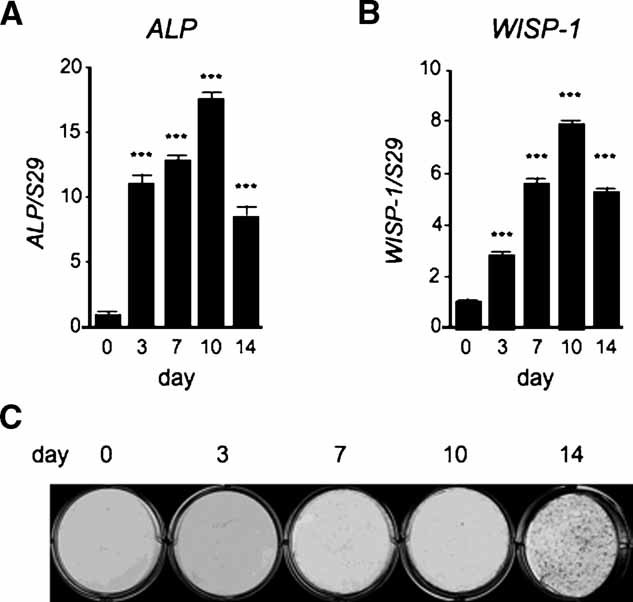
Relative expression of alkaline phosphatase and WISP-1 relative to alizarin red accumulation during the osteogenic differentiation of hBMSCs. Cells were cultured with osteogenic induction medium, and total RNA was collected at 0, 3, 7, 10, and 14 days, and the mRNA expression levels of *ALP* (*A*) and *WISP1* (*B*) were evaluated by real-time PCR. The time points are the numbers of days after the cells reached confluence. The expression of each gene was normalized to that of its respective *S29* ribosomal RNA. Data are reported as mean ± SE (*n* = 3). ***p < .001 versus day 0. (*C*) Alizarin red S staining in the cells whose *ALP* and *WISP1* mRNA expression patterns are shown in panel *A*.

To understand the role of WISP-1 during osteogenesis in vitro, we first determined the mRNA expression profile of *WISP1* in human bone marrow stromal cells (hBMSCs) cultured under conditions conducive for osteogenic differentiation by real-time PCR. Expression of *WISP1* was compared with the expression of the early osteogenic marker alkaline phosphatase (*ALP)* and with calcium accumulation, determined by alizarin red staining, a functional assay of more mature osteoblastic differentiation (Fig. 1). *WISP1* displayed a temporal expression pattern that followed a similar distribution to *ALP* (Fig. 1*A, B*), with peak levels of *WISP1* and *ALP* mRNA expression preceding the onset of calcification (Fig. 1*C*).

### WISP-1 enhances BMP-2 induced osteogenesis in vitro and in vivo

To determine the effects of WISP-1 on in vitro osteogenesis, we generated adenovirus that would express human WISP-1 under the control of the CMV promoter (adWISP-1). We chose to use adenovirus for WISP-1 overexpression studies based on previous work from our laboratory that showed that nearly 100% gene transfer could be achieved in hBMSCs by transduction with adenovirus.(50) First, adWISP-1 was transduced into cultures of hBMSCs, and expression was analyzed by Western blotting. The amount of WISP-1 protein expression was increased substantially compared with cells transduced with control virus (adCMV) in a dose-dependent manner (Supplemental Fig. S1).

**Fig. 2:**
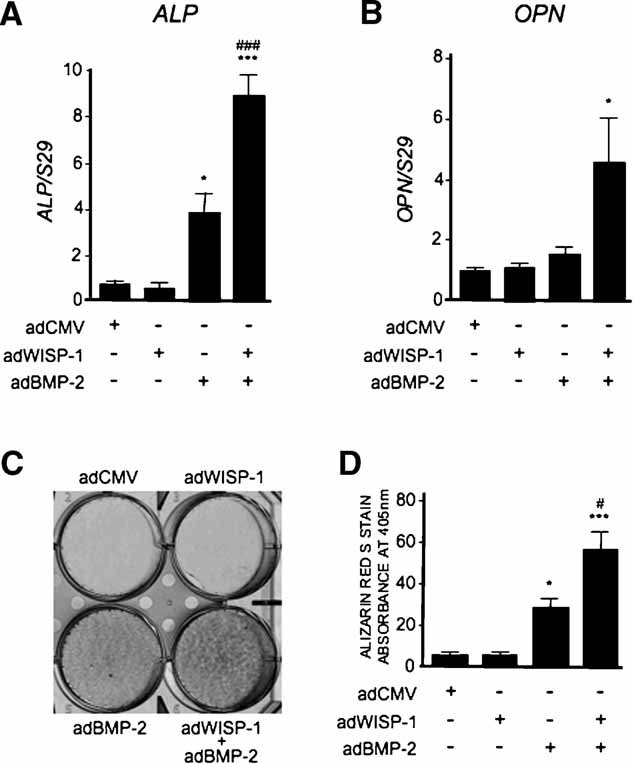
WISP-1 enhances the effects of BMP-2-induced osteoblastic differentiaion in vitro. Relative mRNA expression levels of *ALP* (*A*) and *OPN* (*B*) measured by real-time RT-PCR 7 days after the transduction with adWISP/adBMP-2. The expression of each gene was normalized relative to *S29* ribosomal RNA. *p < .05; ***p < .001 versus adCMV-transduced sample; and ^###^*p* < .001 versus adBMP-2-transduced sample. (*C*) hBMSCs stained with alizarin red S to measure Ca^2+^ deposition after transduction with adWISP-1 and adBMP-2 separately or in combination when cultured in osteogenic induction medium for 2 weeks. (*D*) Quantitative evaluation of alizarin red S staining. Data are reported as mean ± SE (*n* = 3). *p < .05; ***p < .001 versus the sample transduced with adCMV; and ^#^*p* < .05 versus the sample transduced with adBMP-2. AdWISP-1 alone had little effect on hBMSC osteogenic potential but did enhance the effect of adBMP-2 on *ALP* and *OPN* expression (*A*, *B*) and mineralization (*C*, *D*).

To establish how WISP-1 could induce osteogenic differentiation of hBMSCs, we tested the effect of WISP-1 overexpression on BMP-2-induced osteogenic differentiation using adenovirus encoding BMP-2 (adBMP-2). hBMSCs were transduced with either adWISP-1 and adBMP-2 alone or in combination, and after culture in osteogenic differentiation medium, cultures were assayed for expression of osteogenic markers by real-time PCR. After 7 days of culture transduction with adWISP-1 alone, there was no significant effect on osteogenic marker expression. However, adBMP-2-transduced cells had increased osteogenesis and showed significantly higher levels of mRNA for *ALP* (Fig. 2*A*), but not for the later osteogenic marker osteopontin (*OPN)* (Fig. 2*B*). Cotransduction with adBMP-2 and adWISP-1 significantly enhanced *ALP* mRNA levels over and above that of adBMP-2 alone and additionally increased expression of *OPN* (Fig. 2*A, B*). We also tested the effect of WISP-1 overexpression on BMP-2-induced calcium accumulation. As shown by others previously, transduction of hBMSCs with adBMP-2 significantly increased the intensity of alizarin red staining (Fig. 2*C*, *lower left*, *D*, *third bar*), which we could increase further by cotransduction with adWISP-1 (Fig. 2*C*, *lower right*, *D*, *last bar*). When this experiment was repeated using recombinant proteins, the combined application of rhWISP-1 and rhBMP-2 also stimulated osteogenesis (Supplemental Fig. S2), but in our hands it was more variable in outcome than adenovirus transduction. We believe this may be due, in part, to lot variation in the BMP-2, WISP-1, or both. For this reason, we performed the majority of experiments using the adenoviral system. All our data so far support the concept that WISP-1 acts to enhance BMP-2-mediated osteogenesis of hBMSCs.

**Fig. 3:**
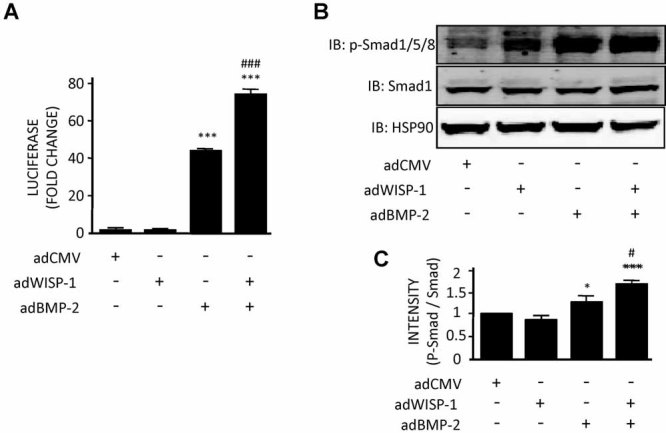
Effect of WISP-1 and BMP-2 on reporter activation and induced Smad-1/5/8 phosphorylation. (*A*) To test the BMP-sensitive Id1 promoter activation, C2C12 cells were transduced with adWISP-1 and adBMP-2 (1000 particles/cell) separately or in combination, and after 5 days, luciferase activity was measured. ***p < .001 versus the sample tranduced with adCMV and ^###^*p* < .001 versus the sample transduced with adBMP-2. (*B*) Smad-1/5/8 phosphorylation was measured in hBMSCs transduced with adWISP-1 and adBMP-2 (5000 particles/cell) separately or in combination. After 48 hour, total cell lysates were collected and resolved by gel electrophoresis and transferred to PVDF membrane and then were incubated with an antibody to either phosphorylated Smad-1/5/8 (p-Smad-1/5/8) or total Smad-1. HSP90 served as an internal control. The experiment was repeated three times, and relative levels of p-Smad/total Smad were plotted graphically in panel *C*. Data are reported as mean ± SE (*n* = 3). *p < .05; ***p < .001 versus the sample transduced with adCMV; and ^#^*p* < .05 versus the sample transduced with adBMP-2.

To determine whether WISP-1 potentiation of BMP-2-induced osteogenic differentiation was mediated directly by enhancing BMP-2 signaling, we examined the effect of WISP-1 on downstream signaling events. First, we analyzed the effect of WISP-1 on BMP-2-induced gene transcription using a BMP-responsive luciferase reporter construct stably transfected into C2C12 cells (C2C12-Bre-Luc).(46) C2C12-Bre-Luc cells were transduced with adWISP-1, adBMP-2, a combination of adWISP-1 and adBMP-2, or a control adenovirus adCMV, and 5 days after transduction, luciferase activity was measured. Luciferase activity was increased 46-fold in adBMP-2-transduced cells compared with cells infected with adCMV (Fig. 3*A*, *bar 3*). C2C12 cells transduced with the combination of adBMP-2 and adWISP-1 had a 78-fold increase in luciferase activity compared with adCMV-transduced cells, WISP-1 thus increasing BMP-2-induced transcription activation almost twofold (Fig. 3*A*, *bar 4*). We next dissected the molecular mechanism involved in WISP-1 enhancing the effect on BMP-2 by analyzing the effect of WISP-1 on BMP-2-induced Smad phosphorylation after 48 hours of transduction. AdBMP-2 transduction increased relative levels of pSmad-1/5/8, but no effect of adWISP-1 transduction was observed, similar to our observations in osteogenic differentiation. However, transduction with both adBMP-2 and adWISP-1 enhanced Smad-1/5/8 phosphorylation over adBMP-2 alone (Fig. 3*B*) in a statistically significant fashion (Fig. 3*C*), suggesting that WISP-1 potentiates BMP-2 activity by increasing the level of Smad-1/5/8 activation. These data indicate that WISP-1 enhances BMP-2 function by increasing Smad-1/3/5 phosphorylation and subsequently activating BMP-2-responsive gene transcription.

**Fig. 4:**
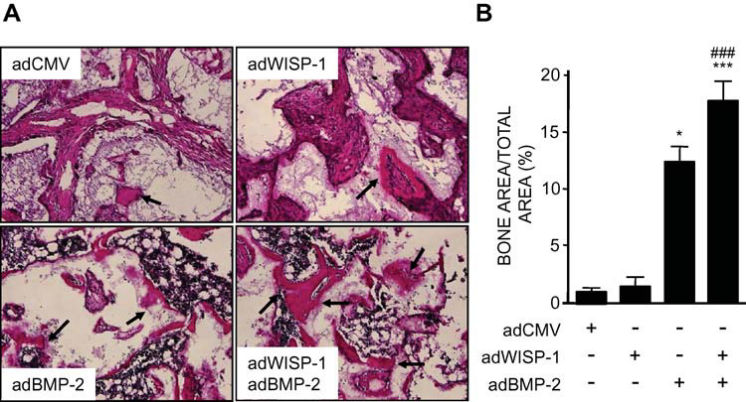
Effects of WISP-1 and BMP-2 on osteogenesis in vivo. hBMSCs were transduced with control CMV adenovirus (adCMV), adWISP-1, adBMP-2, or both and then implanted under the skin of immunocompromised mice. Implants were harvested after 4 weeks and analyzed by histology. (*A*) Representative sections from implants, with black arrows pointing to regions of ectopic bone formation. (*B*) Quantitation of the area of bone formed in four random sections from implants shown in panel *A*. *p < .05; ***p < .001 versus sample transduced with adCMV; and ^###^*p* < .05 versus sample transduced with adBMP-2.

To confirm that our in vitro findings could be replicated in vivo, we performed two different experiments that used either a gain or loss of WISP-1 function strategy. First, in a gain-of-function approach, hBMSCs were transduced with adWISP-1 and adBMP-2 singly or in combination and then implanted under the skin of immunocompromised mice. Four weeks after implantation, the ectopic new bone that was formed by the cells was assessed by histology. As predicted from our in vitro work, adBMP-2-transduced cells generated more bone than cells that were transduced with adCMV (Fig. 4*A, B*). Cells transduced with both adBMP-2 and adWISP-1 had significantly greater bone formation than those transduced with adBMP-2 alone (Fig. 4*A, B*), confirming our earlier in vitro finding that showed that WISP-1 can enhance the ability of BMP-2 to stimulate osteogenesis.

**Fig. 5:**
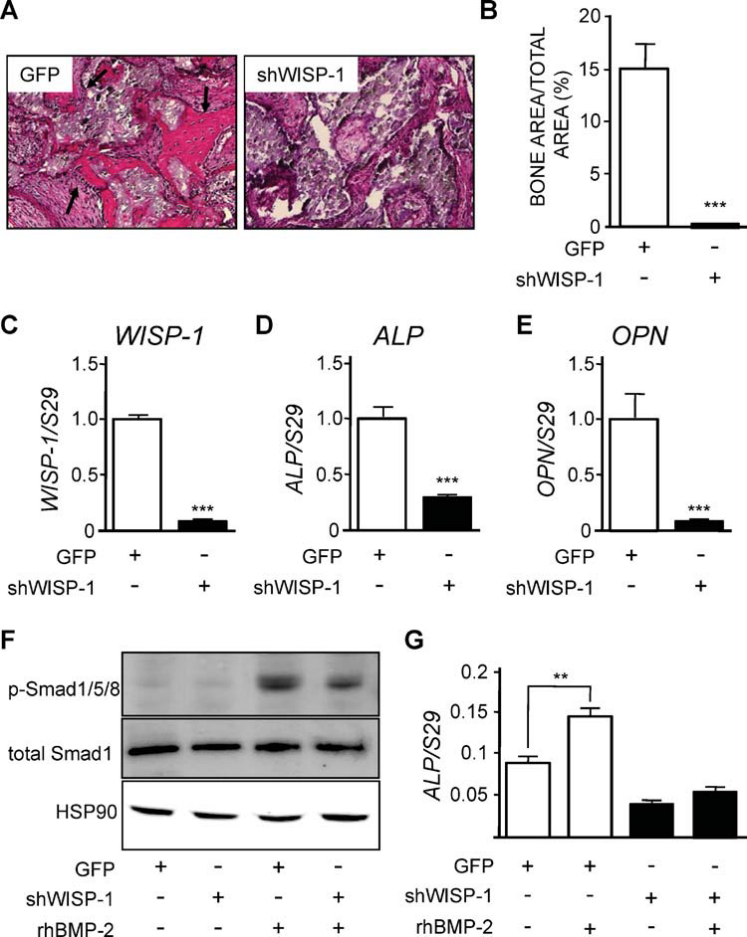
Effect of *WISP1* knockdown on osteogenesis in vivo and in vitro. (*A*) hBMSCs were infected with lentivirus harboring control shRNA (GFP) or shWISP-1 and implanted ectopically to assess bone formation capacity in vivo when harvested at 6 weeks. Arrows point to sites of ectopic bone formed in the implant. (*B*) Quantitation of the area of new bone formed in control lentivirus (GFP) compared with shWISP-1 lentivirus–infected hBMSCs. ***p < .001 versus GFP-infected cells. Relative expression levels of *WISP1* mRNA (*C*), *ALP* mRNA (*D*), and *OPN* mRNA (*E*) in hBMSCs infected with lentivirus containing shRNA to *WISP1*. ***p < .001 versus GFP. (*F*) Relative levels of p-Smad and total Smad-1/5/8 in hBMSCs infected with GFP lentivirus controls and shWISP-1 lentivirus either with or without treatment with recombinant human BMP-2 (rhBMP-2). (*G*) Relative expression of *ALP* mRNA in the same samples shown in panel *F*. **p < .01 versus GFP. *WISP1* shRNA–infected cells showed diminished response to rhBMP compared with control cells.

Our second strategy to assess WISP-1 function in bone used *WISP1* shRNAs packaged into lentivirus. We used lentivirus because of its ability to integrate into host cells and thereby maintain the *WISP1* knockdown throughout the process of osteogenic induction in vitro and in vivo. In this experiment, five different shRNAs were first tested for their ability to knock down *WISP1* expression, and one candidate was chosen, B10, that reduced *WISP1* mRNA levels over 90% compared with controls (Supplemental Fig. S3 and Fig. 5*C*). When tested in vivo using the implant system, hBMSCs infected with *WISP1* shRNA lentivirus had dramatically reduced bone formation compared with cells transduced with control lentivirus (Fig. 5*A, B*) 6 weeks after implantation. This reduction in bone formation appeared to be due to a diminished ability of the *WISP1* shRNA–infected hBMSCs to undergo differentiation, judged by the reduction in the expression of both early and late markers of osteogenesis, *ALP* and *OPN*, respectively (Fig. 5*D, E*). We next used the shRNA system to assess the dependence of BMP-2 function on WISP-1. hBMSCs that had WISP-1 reduced by shRNA showed reduced phosphorylation of p-Smad-1/5/8 in response to adBMP-2 (Fig 5*F*) and, further, had less osteogenic differentiation in response to adBMP-2, judged by the expression of *ALP* mRNA (Fig. 5). It is not clear why overexpression of WISP-1 by adWISP-1 transduction did not significantly affect osteogenesis, whereas in the shRNA *WISP1* knockdown experiment it was decreased significantly, but we presume that factors such as gene transfer mode (lenti versus adeno), timing, or other downstream elements are involved. In addition to that, it must be noted that there was some variability in the osteogenic response of exogenous recombinant BMP-2 depending on the batch used (data not shown). Nevertheless, taken together, these data indicate that the induction of osteogenesis by BMP-2 is enhanced by and depends on the presence of WISP-1.

### WISP-1 directly binds to BMP-2 and can increase BMP-2 binding to hBMSCs

**Fig. 6:**
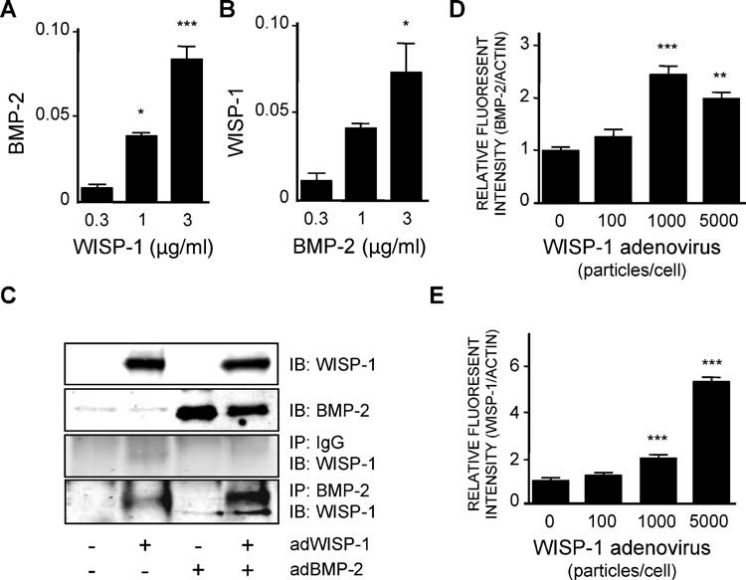
Interaction between WISP-1 and BMP-2. (*A*, *B*) Solid-phase binding assay for WISP-1 and BMP-2. Tissue culture wells were precoated with different concentrations of either WISP-1 (*A*) or BMP-2 (*B*) (0.3, 1, and 3 µg/mL) and incubated with BMP-2 (*A*) or WISP-1 (*B*), respectively, at 37°C for 2 hours. Total binding of each protein was determined by measuring immunoreactivity toward the anti-BMP-2 antibody (*A*) or anti-WISP-1 antibody (*B*). Data are reported as mean ± SE (*n* = 4). *p < .05; ***p < .001 versus the sample treated with 0.3 µg/mL of protein. (*C*) Immunoprecipitation using BMP-2 antibody. hBMSCs were transduced with adWISP-1 and adBMP-2 separately or in combination, and after 3 days, cell lysates were collected. Immunoprecipitation was performed using an anti-BMP-2 antibody, and the amount of WISP-1 “pulled down” was measured using an anti-WISP-1 antibody. (*D*) The affinity of BMP-2 for binding to hBMSCs transduced with adWISP-1. hBMSCs were transduced with adWISP-1 in hBMSCs, and 3 days later, the cells were fixed and incubated with BMP-2 for 2 hours. The amount of BMP-2 bound to the cells was evaluated by measuring the amount of immunoreactivity using an anti-BMP-2 antibody. The relative amount of BMP-2 present was normalized to the levels of β-actin. Data are reported as mean ± SE (*n* = 4). **p < .01; ***p < .001 versus the nontransduced sample. (*E*) Levels of cell surface associated WISP-1 in hBMSCs transduced with adWISP-1, and after 3 days, the amount of WISP-1 was normalized to β-actin. Data are reported as mean ± SE (*n* = 4). ***p < .001 versus the nontransduced sample.

Previous studies have shown that other CCN family members can bind to BMPs and regulate their function.(15,16) Analysis of WISP-1 protein structure by our laboratory and others has indicated a region in the CT domain with high homology to the BMP-2 antagonist noggin(11) and another cysteine-rich region homologous to chordin in the VWF-like domain.(13) Given this evidence and our functional data indicating that WISP-1 can affect BMP-2 action, we speculated that WISP-1 may bind to BMP-2 to regulate its function. To test this, we used solid-phase binding and coimmunoprecipation experiments. When increasing amounts of WISP-1 were coated onto tissue culture plates and then incubated with BMP-2, the amount of BMP-2 detected using BMP-2-specific antibody increased proportionally to WISP-1 concentration (Fig. 6*A*). In the reverse, when increasing amounts of BMP-2 were coated onto solid supports and were incubated subsequently with WISP-1, we found that the amount of WISP-1 detected using antibodies to WISP-1 also increased proportionally to the amount of BMP-2 bound to the plate (Fig. 6*B*). A comparison of BMP-2 binding to other members of the CCN family revealed that BMP-2 had greater binding affinity for WISP-1 than for CTGF but lower than that for Nov (Supplemental Fig. S4). To determine whether WISP-1 and BMP-2 could bind directly to each other in cells, we next performed coimmunoprecipiation experiments (co-IP) in which hBMSCs were transduced with adWISP-1 and/or adBMP-2, and then 5 days later, cell lysates were subjected to co-IP. The specificity of the experiment was tested using both positive controls for WISP-1 and BMP-2 (IB: WISP-1; IB: BMP-2) and negative controls using lysates immunoprecipiated with antibody to IgG (IP: IgG). Western blot analysis for WISP-1 of cell lysates immunoprecipitated with a BMP-2 antibody revealed that the BMP-2 immunoprecipitate contained WISP-1 (Fig. 6*C*, *bottom panel*, IP: BMP-2, IB: WISP-1), indicating an interaction between WISP-1 and BMP-2 under these conditions. Some BMP-2 was observed immunoprecipitated in samples without prior adBMP-2 treatment and was assumed to come from endogenous BMP-2 constituatively produced by the hBMSCs. WISP-1 and BMP-2 are both secreted proteins, and thus interactions must take place outside the cell. Therefore, we next tested whether WISP-1 overexpression could affect BMP-2 binding to hBMSCs. Cultures transduced with increasing doses of adWISP-1 were incubated with BMP-2 for 2 hours and then assayed using antibodies against BMP-2. As a control, WISP-1 levels a were also assayed using anti-WISP-1 antibody. Increasing amounts of BMP-2 were detected paralleling increased transduction with adWISP-1 (Fig. 6*D*), and increased expression of WISP-1 with maximum effects observed at 1000 PFU/cell (Fig. 6*E*). It is not clear why cells having the highest levels of WISP-1 had slightly less BMP-2 bound, but we assume that this may be from yet to be identified downstream effects on the treated cells. These data taken together support the concept that WISP-1 can enhance BMP-2 function by facilitating its binding to the surface of hBMSCs and induce downstream signaling and transcription of BMP-2-sensitive genes, thereby enhancing BMP-2-induced osteogenic differentiation.

### Integrin α_5_ β_1_ may regulate the functional relationship of WISP-1 and BMP-2

**Fig. 7:**
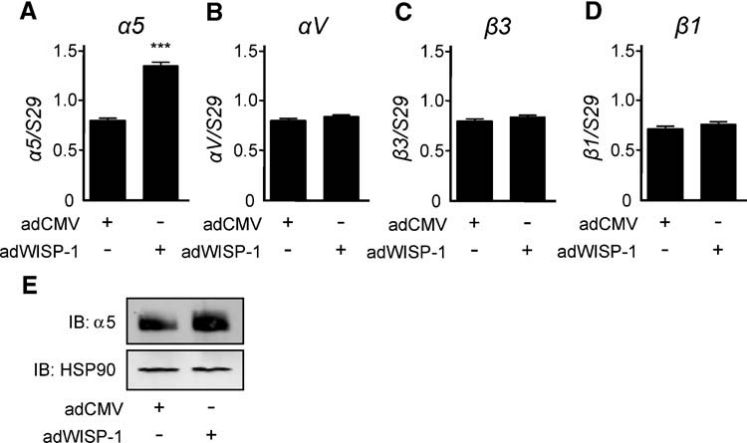
Effect of WISP-1 on the production of α_5_ . (*A–D*) Relative expression levels of *α_5_*, *α_V_*, *β_3_*, and *β_1_* mRNA in hBMSCs transduced with adWISP-1 or adWISP-1 for 3 days, as determined by real-time PCR. The expression of each gene was normalized to the level of *S29* ribosomal RNA. ***p < .001 versus the sample transduced with adCMV. (*E*) Representative Western blot analysis of α_5_ protein in hBMSCs transduced with adWISP-1 for 3 days.

Previous studies have indicated that the CCN family can act to regulate the expression of integrins, and in turn, integrins can serve as receptors for CCN family members such as Cyr61.(31,32,34–38,51) We determined the effect of WISP-1 on the expression of mRNA encoding α_5_, α_V_, β_3_, and β_1_, integrin subunits shown previously to be regulated by CCN family members, in hBMSCs. No effects on the expression level of *α*_*V*_, *β*_*3*_, or *β*_*1*_ mRNA levels were observed following adWISP-1 transduction (Fig. 7*B–D*). However, we observed significant effects of adWISP-1 transduction on integrin *α*_*5*_ mRNA that was increased almost twofold (Fig. 7*A*) compared with adCMV-transduced controls. These increases in *α*_*5*_ mRNA levels were confirmed at the protein level by Western blotting (Fig. 7*E*), indicating that overexpression of WISP-1 selectively increases *α*_*5*_ mRNA and protein in hBMSCs.

**Fig. 8:**
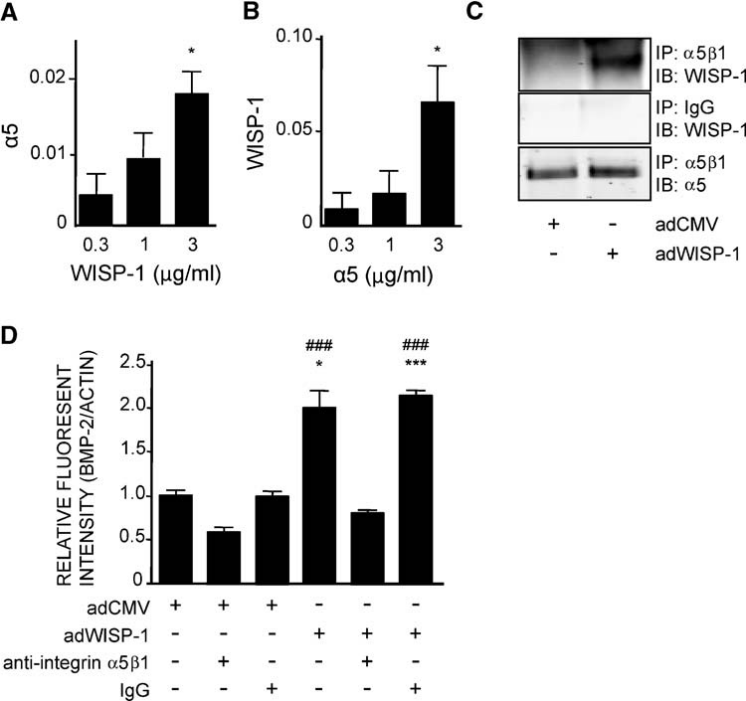
Interaction of WISP-1 with α_5_ β_1_ . (*A*, *B*) Solid-phase binding of WISP-1– integrin α_5_ β_1_ interactions. Tissue culture plates were coated with different concentrations of WISP-1 (*A*) or integrin α_5_ β_1_ (*B*) (0.3, 1, and 3 µg/mL) and were incubated with integrin α_5_ β_1_ (*A*) or WISP-1 (*B*), respectively, at 37°C for 2 hours. The amount of binding of each protein was determined by measuring immunoreactivity toward the anti-integrin α_5_ β_1_ antibody (*A*) or anti-WISP-1 antibody (*B*). Data are reported as mean ± SE (*n* = 4). *p < .05 versus the sample treated with 0.3 µg/mL of protein. (*C*) Western blotting showing that WISP-1 binds to integrin α_5_ β_1_ using immunoprecipitation with anti-integrin α_5_ β_1_ antibody. hBMSCs were transduced with adWISP-1 (5000 particles/cell), and 3 days later, the cell lysates were collected. Then immunoprecipitation and Western blotting were performed with anti-integrin α_5_ β_1_ antibody and anti-WISP-1 antibody, respectively. (*D*) Inhibition assay using anti-integrin α_5_ β_1_ antibody. hBMSCs, were preincubated with or without anti-integrin α_5_ β_1_ antibody (10 µg/mL) or IgG antibody (10 µg/mL) for 1 hour, and then cells were transduced with adWISP-1 (5000 particles/cell). Three days after transduction, the cells were fixed and incubated with BMP-2 for 2 hours. BMP-2^+^ cells were detected by measuring immunoreactivity toward the anti-BMP-2 antibody. The graph shows the quantitation of these data normalized to β-actin. Data are reported as mean ± SE (*n* = 4). *p < .05; ***p < .001 versus nontreatment in the sample transduced with adCMV; ^###^*p* < .001 versus treatment with anti-integrin α_5_ β_1_ antibody in the sample transduced with adWISP-1.

To investigate the significance of WISP-1's selective effect on α_5_ subunit expression, we first tested the possibility that there is a physical interaction between WISP-1 and the α_5_ subunit by solid-phase binding assay and co-IP. Solid-phase binding revealed that the amount of α_5_ β_1_ detected depended on the amount of WISP-1 protein present (Fig. 8*A*). Conversely, the amount of bound WISP-1 detected was increased dose-dependently by the amount of integrin α_5_ β_1_ protein present (Fig. 8*B*). This interaction was confirmed in vitro by co-IP of cells transduced with adWISP-1 for 5 days, revealing that IP with antibodies to α_5_ β_1_ could pull down WISP-1 that was detected by Western blot analysis (Fig. 8*C*). Finally, to determine if there is a functional relationship between WISP-1, BMP-2, and integrin α_5_ β_1_, we performed an inhibition assay using α_5_ β_1_ -blocking antibodies. In this experiment, hBMSCs were transduced with adWISP-1 or adCMV control, and then BMP-2 binding was tested in the presence or absence of blocking antibody to α_5_ or to the control IgG. As shown in Fig. 8*D*, the amount of BMP-2 bound to cells that overexpressed WISP-1 was inhibited by the addition of antibodies to integrin α_5_ . Treatment with IgG control antibody did not diminish the binding of BMP-2 to WISP-1-overexpressing cells, indicating that the effect was specific for the interaction with α_5_ . Cells treated with blocking antibodies to α_5_ β_1_ had total inhibition of osteogenesis judged by *ALP* mRNA expression (Supplemental Fig. S5), and while revealing the importance of α_5_ β_1_ in *ALP* mRNA production, it precluded further testing of WISP-1 in the differentiation process. Nevertheless, taken together, these results suggest that WISP-1 potentially could enhance BMP-2 binding to BMSCs through an interaction involving integrin α_5_ β_1_ .

### Pilot characterization of *WISP1* transgenic mice

**Fig. 9:**
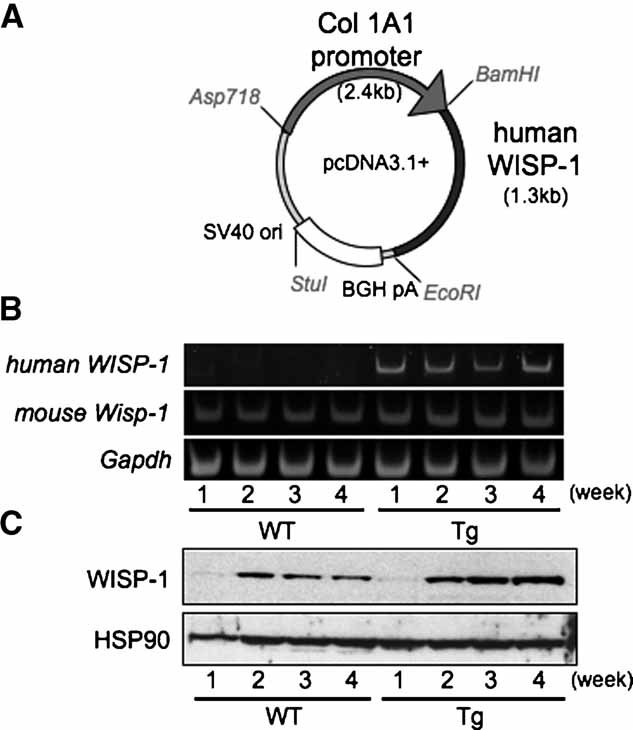
The expression of hWISP-1 in transgenic mice designed to express hWISP-1 in bone. (*A*) A 1.3-kb DNA fragment coding for human WISP-1 was cloned downstream of a 2.4-Kb DNA containing the Col1A1 bone-specific promoter (2.3-kb promoter plus 0.1-kb exon 1) and was used to make transgenic mice. (*B*) mBMSCs were collected from *WISP1-Tg* and wild-type mice and cultured with osteogenic induction medium for up to 4 weeks after confluence. The relative expression levels of human *WISP1* mRNA were determined in mBMSCs derived from wild-type (*left*) or *WISP1-Tg* (*right*) mice. In order to detect human *WISP1* and mouse *WISP1*, RT-PCR was performed using human WISP-1 or mouse-specific primers, respectively (Table 1). RT-PCR of mRNA encoding *Gapdh* served as an internal control. (*C*) To analyze the relative levels of WISP-1 protein, a Western blot was performed using anti-WISP-1 antibody (*upper panel*), where HSP90 served as an internal control (*lower panel*). The antibody, LF-187, crossreacted with mouse WISP-1 but showed higher total levels of WISP-1 (human plus mouse) in the *WISP1-Tg* cells as they became more osteogenic.

To further confirm that there are potential functions for WISP-1 during osteogenesis in vivo and to create an animal model for future studies, a transgenic mouse line was generated that expressed WISP-1 under the control of the 2.3-kb Col1Al promoter, which directs expression to mineralized tissues (Fig. 9*A*). At birth, no gross abnormalities were observed, and normal Mendelian ratios were observed; moreover, the overall health of these transgenics (termed *WISP1-Tg*) appeared to be good. However, we found that female transgenics could not give birth, and therefore, the lines could be maintained only by breeding male *WISP1-Tg* mice with wild-type female mice. Of the six founders identified by Southern blot analysis, *WISP1-Tg* line 5 was chosen for detailed analysis. This line was shown to significantly express the human mRNA transgene, judged by the amount of RNA amplified from osteoprogenitor cells using human-specific oligonucleotides for RT-PCR (Fig. 9*B*). Western blotting of cell extracts from the same cells was performed using an antibody (LF-187) generated from a conserved amino acid sequence that recognizes both mouse and human WISP-1 and found the level of expression of WISP-1 to be increased above endogenous mouse WISP-1 levels in BMSCs from *WISP1-Tg* mice compared with the wild-type mice (Fig. 9*C*).

**Fig. 10:**
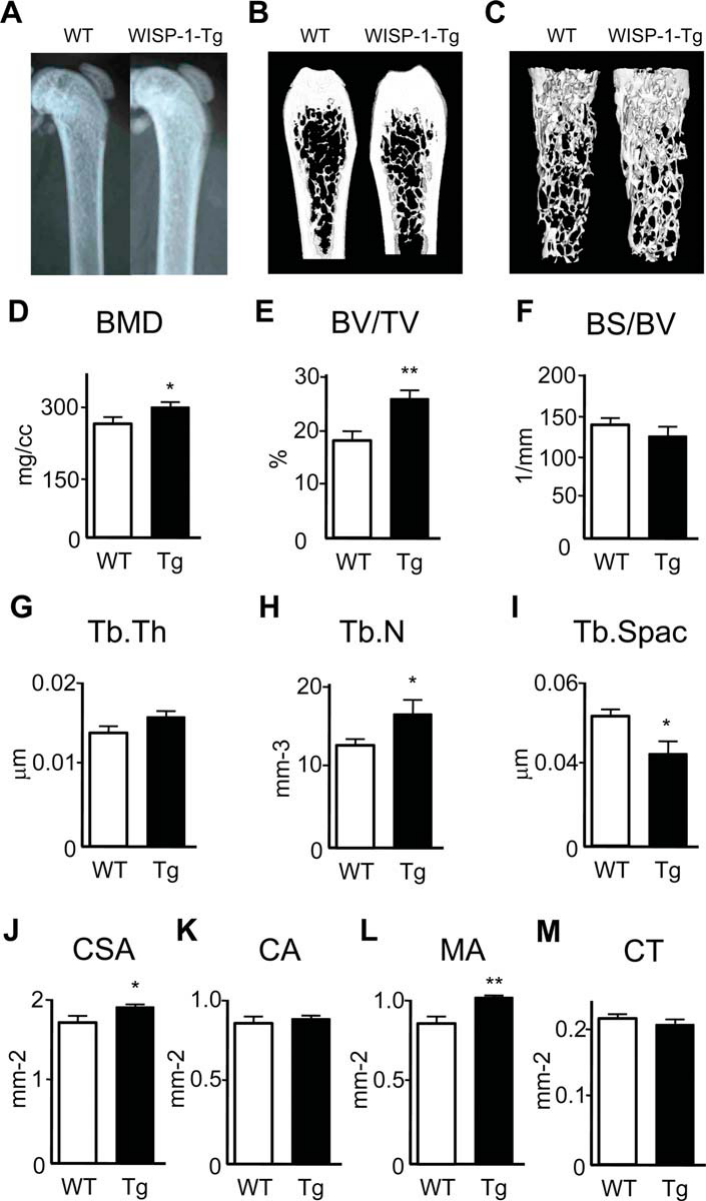
Bone phenotype of the *WISP1-Tg* mice. (*A*) X-ray images of the femurs from 8-week-old *WISP1-Tg* and wild-type mice revealing increased density of bone, depicted by increased X-ray intensity. (*B*) µCT images of of wild-type and *WISP1-Tg* mice, confirming increased bone density, as shown graphically by 2D images of sagittal sections of femurs from 8-week-old wild-type and *WISP1-Tg* mice. (*C*) 3D rendering of the trabecular bone shown. (*D*) Trabecular bone mineral density (BMD), (*E*) trabecular bone volume per tissue volume (BV/TV), (*F*) bone surface per bone volume (BS/BV), (*G*) trabecular thickness (Tb.Th), (*H*) trabecular number (Tb.N), and (*I*) trabecular spacing (Tb.S), (*J*) cross-sectional area (CSA, mm^2^), (*K*) cortical area (CA, mm^2^), (*L*) medullary area (MA, mm^2^), (*M*) cortical thickness (mm). All measurements were obtained either from an elliptical cylinder within the metaphases or by imaging a cross-sectional area in the midshaft region of the femur. Data are reported as mean ± SE (*n* = 6). *p < .05; **p < .01 versus wild-type sample.

We first analyzed the skeleton of transgenic mice by X-ray analysis, revealing that the most significant effects of WISP-1 were observed in the long bones of the axial skeleton. X-ray of extracted long bones from 3-month-old wild-type and *WISP1-Tg* mice revealed that transgenic mice had denser bone than wild-type mice, and this was effect was most pronounced in *WISP1-Tg* females (Fig. 10*A*). µCT 3D reconstruction of femurs from 2-month-old females confirmed our observations of increased bone density and, further, indicated that the trabecular bone was the most affected area (Fig. 10*B, C*). Finer analysis of the distal femurs revealed that the bone mineral density (BMD; Supplemental Fig. S7*D*), bone volume/total volume (BV/TV; Fig. 10*E*), and trabecular number (Tb.N; Fig 10*H*) were significantly higher, and correspondingly, trabecular spacing (TB.Spac) was significantly lower (Fig. 10*I*) in the *WISP1-Tg* mice than in the wild-type mice. While the trabecular thickness (Tb.Th) and the bone surface/bone volume (BS/BV) showed an increased and decreased trend, respectively, in the *WISP1-Tg* mice compared with the wild-type mice, they were not significantly different (Fig. 10*F*). The cross-sectional area (CSA; Fig. 10*J*) and medullary area (MA; Fig. 10*L*) also were increased in the *WISP1-Tg* mice compared with the wild-type mice, but no significant differences were observed cortical area (CA; Fig. 10*K*) or in cortical thickness (CT; Fig. 10*M*). It must be clearly noted that that these data are reamed from a single transgenic line and will need to be confirmed with additional lines of mice or by creating *WISP1* knockout mice. With this caveat in mind, these data provide another line of evidence to support the idea that WISP-1 has a positive effect on bone formation in vivo (Fig. 5*A*).

## Discussion

The goal of this study was to determine the role of WISP-1 in osteogenesis and to further understand its mechanism of action. The results presented in this article indicate that WISP-1 can act to induce osteogensis by enhancing the ability of BMP-2 to induce osteoblastic differentiation of osteoprogenitor cells in the bone marrow. The molecular basis of the cooperative action of WISP-1 and BMP-2 appears to work through an interaction of integrin α_5_ β_1_ at the cell surface of BMSCs. Pilot studies using one line of transgenic mice further indicate that WISP-1 has a positive influence on bone formation in vivo.

Our previous work indicated that WISP-1 might act cooperatively with members of the TGF-β superfamily to regulate osteoblast function. BMPs are members of the TGF-β superfamily that can regulate numerous cell activities, including differentiation, proliferation, and apoptosis.(42,43) As their name implies, they play important roles in regulating the development and maintenance of skeletal tissues.(52,53) During osteogenesis, cells express a series of proteins that are characteristic of the differentiation process and include the production of Runx-2, alkaline phosphatase, bone sialoprotein (BSP), and osteocalcin (Ocn).(54) This osteogenic process can be regulated by several members of the BMP family, including BMP-2, BMP-4, and BMP-7.(42,43) During later stages of differentiation, antagonists of BMP such as noggin and chordin have important functions such that osteogenesis ultimately is controlled by a fine balance between the levels of BMPs and their antagonists. The expression of WISP-1 in preosteogenic cells is quite distinct from other CCN proteins that are more prominently associated with cartilage development at sites of secondary ossification.(55) This observation indicates that the function of CCN2 and CCN3 in bone might be distinct from CCN4/WISP-1. Evidence to support this notion comes from the fact that mice that overexpress CCN2/CTGF or CCN3/Nov in bone have an osteopenic phenotype.(15,16) In contrast, we show here that overexpression of WISP-1 enhances BMP-2-induced osteogenesis. CCN2, CCN3, and CCN4 all have been shown bind to BMP-2. For CCN2/CTGF and CCN3/Nov, this binding appears to inhibit BMP action,(39,56) whereas for CCN4/WISP-1, it appears to enhance it. Thus, despite the apparent sequence similarities among different members of the CCN family, it seems that they can serve as BMP antagonists or agonists depending on the CCN type. This differential action of individual CCN members could be due to their unique temporal-spatial patterns of expression, where functional consequences could be cell-, tissue-, or even age-dependent.

Since there are no known receptors for WISP-1, to further understand the molecular mechanism involved in WISP-1 potentiation of BMP-2 action, we investigated the possibility that WISP-1 may have an impact on pathways that can crosstalk with BMP signaling. We finally focused our attention on the integrins after examination of the ERK, p38, or JNK signaling pathways; other pathways known to converge on BMP-2 signaling were found not to be differentially regulated by the combined application of WISP-1 and BMP-2 (not shown). Integrins have been identified as functional receptors for some members of the CCN family, including CCN1/Cyr61 and CCN2/CTGF.(12,33,36,57–62) In some cases, the CCN proteins interacted with other extracellular matrix proteins such as fibronectin and, in so doing, play important roles in regulating cell adhesion and migration.(36,57–59) Using cells deficient in CCN2, Nishida and colleagues(63) found that the expression level of integrin α_5_ β_1_ was downregulated. On the other hand, the expression of α_5_ β_1_ was increased by adding CCN2 to chondrocytes. Antibody blocking experiments have shown that CCN2 binding to fibronectin was blocked using antibodies to α_5_ β_1_ .(36) A number of studies also have implicated integrins in the activation of signaling via TGF-β superfamily members, and cells lacking in α_5_ β_1_ integrin completely lack the ability to signal via TGF-β1.(64) These studies led us to speculate that integrins also could be involved in the BMP-enhancing functions that we uncovered for WISP-1 using cultured hBMSCs. Our experiments indicate that overexpression of WISP-1 increases the expression of *α*_*5*_ mRNA and protein in BMSCs. The functional consequence of the increased α_5_ was further defined by experiments showing direct protein-protein interactions between WISP-1 and integrin α_5_ β_1_ . The essential role of α_5_ in WISP-1 enhancement of BMP-2 cell binding was revealed using blocking antibodies to this integrin subunit. Support of the importance of α_5_ in priming hBMSC toward osteoblast differentiation and osteogenesis has been reported recently(65); however, it is conceivable that there are additional associating factors that could further regulate the WISP-1/BMP-2/α_5_ complex, but this still remains to be determined. It also must be cautioned that the additional experiments are needed to definitively prove the involvement of α_5_ in WISP-1/BMP-2-induced osteogenesis. It is also possible that receptors such as LRP5/6 could be involved in WISP-1 function in bone in a way that is similar to what has been reported previously for CCN2/CTGF.(66,67) Sclerostin is an essential regulator of bone mass that has been reported to act on both LRP5/6 and BMP-2, and humans with inactivating mutations in sclerostin/*SOST* develop van Buchem disease, which is characterized by significantly increased bone mass.(8,68) Interestingly, sclerostin bears significant homology with the BMP antagonist noggin(69) and is also known act on BMP signaling as well as Wnt signaling, albeit to a significantly lesser extent.(70–72)

Recent reports have suggested there is a relationship between WISP-1 and skeletal disease. For example, mutations in the related *CCN6*/*WISP3* have been found to be the genetic basis the autosomal recessive skeletal disorder called *progressive pseudorheumatoid dyplasia* (PPD).(73) A single-nucleotide polymorphism (SNP) in the *WISP1* gene has been identified that has a strong association with the severity of spinal osteoarthritis (OA) in a population of postmenopausal Japanese women.(74) Recently, Blom and colleagues found that WISP-1 was highly increased in the human OA and in the synovium and cartilage of mice with experimentally induced OA.(9) With regard to this latter study, it is interesting to speculate that one reason WISP-1 might be upregulated during OA is the increased bone turnover that can occur in the subchondral bone in OA diseased tissue.(75) Our finding that WISP-1 can enhance BMP actions further suggests that WISP-1 could contribute functionally to the bone changes noted in the progression of OA.(76) It is not clear at this time which part of WISP-1 binds to BMP, but it is reasonable to speculate that it could be the chordin-like domain known to bind BMP in other CCN family members and in some cases even having enhancing effects.(51)

Our finding that WISP-1 enhances the ability of BMP-2 to stimulate osteogenesis could have important future applications for improving BMP-2 function in bone healing. Initial studies on the use of BMPs for regenerative medicine were extremely promising when used in animals, but when tested in human applications, they proved to be suboptimal and required relatively large doses of the growth factor to be effective (see refs. (44) and (52) for excellent reviews of the history and use of BMPs for orthopedic trauma surgery). In order to improve the performance of BMPs, several investigators have examined the use of multiple BMPs and combinatorial gene/protein therapy. These studies suggest that combinations of BMP-2 and -7(77,78) or intermittent parathyroid hormone (PTH) therapy(79) can improve the efficacy of BMP in repairing bone tissue during fracture repair or to improve healing after craniofacial surgery. Potentially, WISP-1 could be used to enhance BMP-2 function in clinical applications requiring the stimulation of osteogenesis.

In conclusion, our studies show that WISP-1 can enhance the effects of BMP signaling and may work through an interaction with α_5_ β_1_ and that WISP-1 can have an anabolic effect on bone mass in vivo when expressed in bone cells. It is possible that multiple other cell types could be affected by WISP-1, including osteoclasts, which are known to have an intimate relationship with osteoprogenitor cells where WISP-1 is expressed. Future investigations will be designed to identify the precise structural domains in WISP-1 as well as additional accessory factors within the extracellular matrix of bone that may modulate WISP-1 function to control skeletal maintenance with aging.
