# Supplementary material for: WISP-1/CCN4 Regulates Osteogenesis by Enhancing BMP-2 Activity
Source: J Bone Miner Res. 2010 Aug 3;26(1):193–208. doi: 10.1002/jbmr.205 (PMC3179320; doi:10.1002/jbmr.205)
Supplement: Supplementary file 1 [file jbmr0026-0193-SD1.doc]

Supplemental Methods

*Generation of transgenic mice over-expressing human WISP-1*

A plasmid containing a full-length human WISP-1 cDNA was the kind gift from Arnold Levine, The Rockefeller University, NY. 1.3 kb WISP-1 fragment was purified from pBabe-Puro retroviral DNA vector and sub-cloned into pcDNA3.1- (Invitrogen, Carlsbad, CA) using *BamHI* and *EcoRI* sites. Large-scale synthesis and purification of endotoxin-free plasmids was carried out using standard techniques and the Qiagen endo-free maxi preparation purification kit (Qiagen, Gaithersburg, MD) and the integrity of the construct was confirmed by DNA sequencing. The Col1A1 promoter (-2310 to +110) was the kind gift from Benoit deCrombrugge, MD Anderson Cancer Center, TX. Following release from the pJ251 using *Asp718* and *BamH1* sites and the promoter was cloned immediately upstream of WISP-1 in pcDNA3.1- to produce a final construct of 9.12 kb (Col1A1-WISP-1-pcDNA). Transgenic mice that express WISP-1 under control of the Col1AI promoter were generated using a 4.8 kb DNA fragment including the Col1A1 promoter (2.3 kb of promoter 0.1 kb of exon 1), WISP-1 (1.3 kb) and the Bovine Growth Hormone poly A tail (BGH pA) (1.1 kb) which was excised from Col1AI-WISP-1-pcDNA using *ASP718* and *Stu,I* and purified by sucrose gradient. Transgenic mice were prepared by pro-nuclear injection with assistance from the NIDCR-DIR Functional Genomics Core Facility. Six founder mice were identified that contained the transgene as judged by Southern blot analysis of DNA isolated from mouse tails using the 4.8kb fragment generated for pro-nuclear injection as the probe. Two of the founder lines showed high levels of transgene integration. One mouse line that over-expressed the transgene as detected by western blot analysis of mouse bone was identified and was subsequently studied in greater detail. A PCR strategy was devised for routine genotyping using primers in the 3’end of the col1A1 promoter (5’TGGACTCCTTTCCCTTCCTT3’) and the 5’end of the human WISP-1 cDNA (5’GCAGGAACCACCTCATGC3’).

*X-ray and Micro-computed Tomography Analysis*

Femurs were dissected from WT and WISP-1 transgenic mice and subject to radiography using Kodak X-OMAT TL film and a model FS-20 Faxitron X-ray system at a setting 30 kv using a 40-second exposure time. The femurs were scanned and reconstructed with 8 μm isotropic voxels on a micro-computed tomography analysis (μCT) system (eXplore MS, GE Medical Systems, London, Ontario, Canada). A bone standard (SB3, Gammex RMI, Middleton, WI) was scanned with the μCT and used for the calibration of bone-mass measurements. Reconstructed 3D images of distal femurs were analyzed using Microviewer (GE Medical Systems, London, Ontario, Canada). A fixed threshold was used to separate the bone and marrow phase. The trabecular bone mineral density (BMD), trabecular bone volume per tissue volume (BV/TV), Bone surface per bone volume (BS/BV), trabecular thickness (Tb.Th) trabecular number (Tb.N), and trabecular spacing (Tb.Spac) in the distal femur were measured in a rectangular cylinder within the metaphysis. Cortical surface area (CSA), cortical area (CA), marrow area (MA) and cortical thickness (CT) were measured in a rectangular circle in the diaphysis.
