# Supplementary material for: WISP-1/CCN4 Regulates Osteogenesis by Enhancing BMP-2 Activity
Source: J Bone Miner Res. 2010 Aug 3;26(1):193–208. doi: 10.1002/jbmr.205 (PMC3179320; doi:10.1002/jbmr.205)
Supplement: Supplementary file 2 [file jbmr0026-0193-SD2.ppt]

## Slide 1
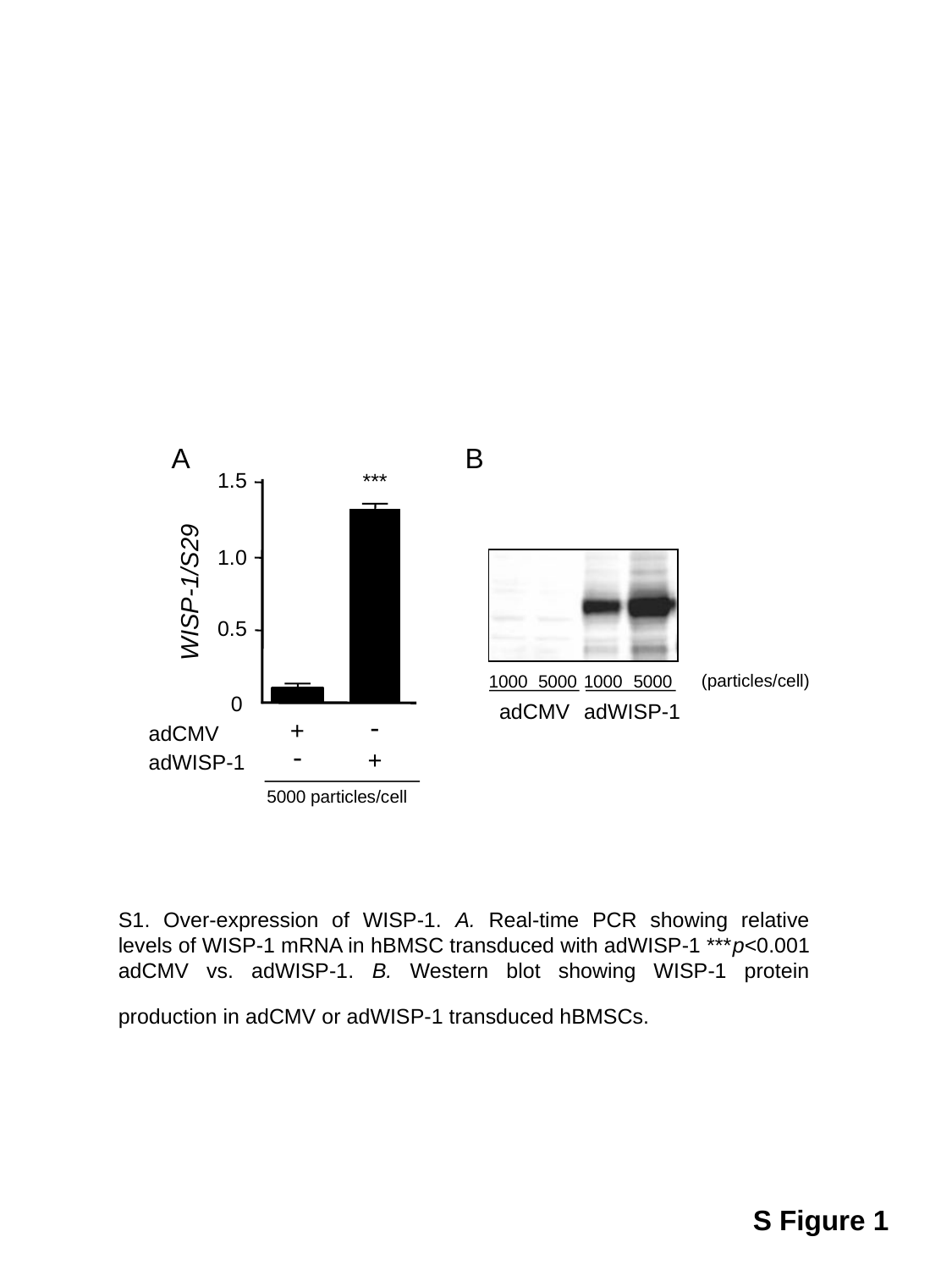

A
B
1.5
***
1.0
WISP-1/S29
0.5
0
-
+
adCMV
-
+
adWISP-1
(particles/cell)
1000
5000
1000
5000
adCMV
adWISP-1
5000 particles/cell
S1. Over-expression of WISP-1. A. Real-time PCR showing relative levels of WISP-1 mRNA in hBMSC transduced with adWISP-1 ***p<0.001 adCMV vs. adWISP-1. B. Western blot showing WISP-1 protein production in adCMV or adWISP-1 transduced hBMSCs.
S Figure 1
