# Supplementary material for: WISP-1/CCN4 Regulates Osteogenesis by Enhancing BMP-2 Activity
Source: J Bone Miner Res. 2010 Aug 3;26(1):193–208. doi: 10.1002/jbmr.205 (PMC3179320; doi:10.1002/jbmr.205)
Supplement: Supplementary file 3 [file jbmr0026-0193-SD3.ppt]

## Slide 1
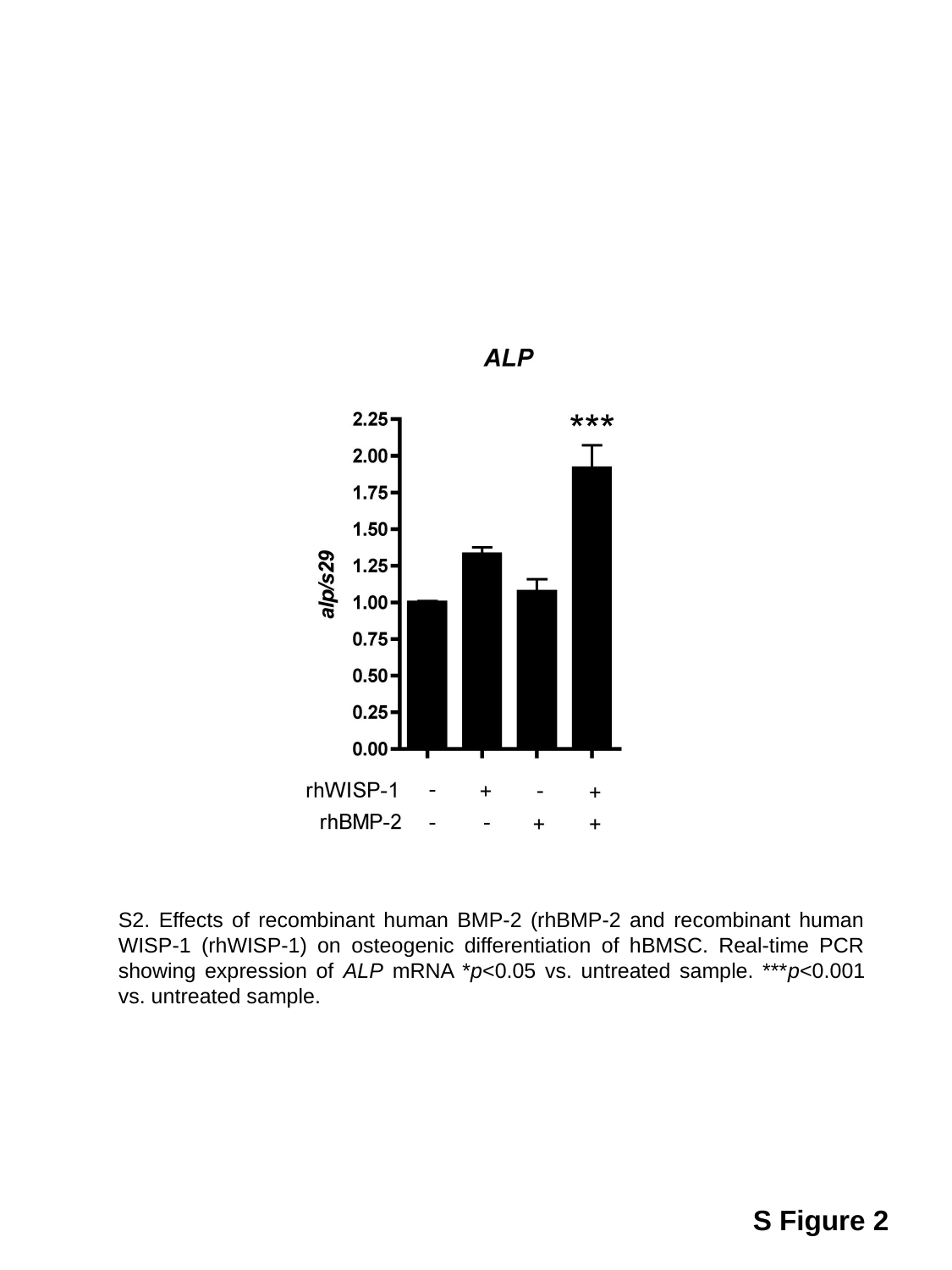

S2. Effects of recombinant human BMP-2 (rhBMP-2 and recombinant human WISP-1 (rhWISP-1) on osteogenic differentiation of hBMSC. Real-time PCR showing expression of ALP mRNA *p<0.05 vs. untreated sample. ***p<0.001 vs. untreated sample.
S Figure 2
