# Supplementary material for: WISP-1/CCN4 Regulates Osteogenesis by Enhancing BMP-2 Activity
Source: J Bone Miner Res. 2010 Aug 3;26(1):193–208. doi: 10.1002/jbmr.205 (PMC3179320; doi:10.1002/jbmr.205)
Supplement: Supplementary file 4 [file jbmr0026-0193-SD4.ppt]

## Slide 1
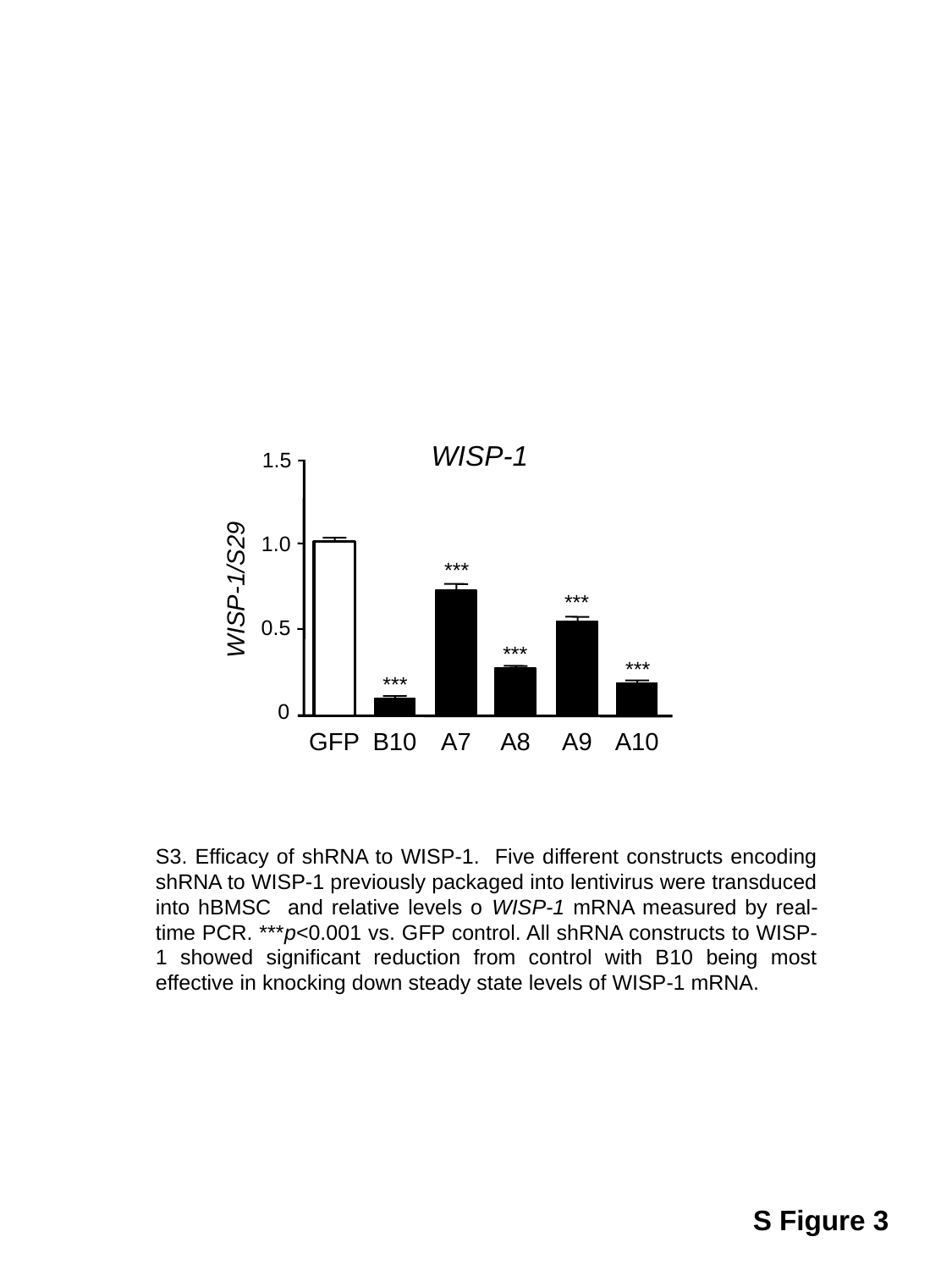

WISP-1
1.5
1.0
***
WISP-1/S29
***
0.5
***
***
***
0
GFP
B10
A7
A8
A9
A10
S3. Efficacy of shRNA to WISP-1. Five different constructs encoding shRNA to WISP-1 previously packaged into lentivirus were transduced into hBMSC and relative levels o WISP-1 mRNA measured by real-time PCR. ***p<0.001 vs. GFP control. All shRNA constructs to WISP-1 showed significant reduction from control with B10 being most effective in knocking down steady state levels of WISP-1 mRNA.
S Figure 3
