# Supplementary figures and images for: WISP-1/CCN4 Regulates Osteogenesis by Enhancing BMP-2 Activity
Source: J Bone Miner Res. 2010 Aug 3;26(1):193–208. doi: 10.1002/jbmr.205 (PMC3179320; doi:10.1002/jbmr.205)

## Slide 1
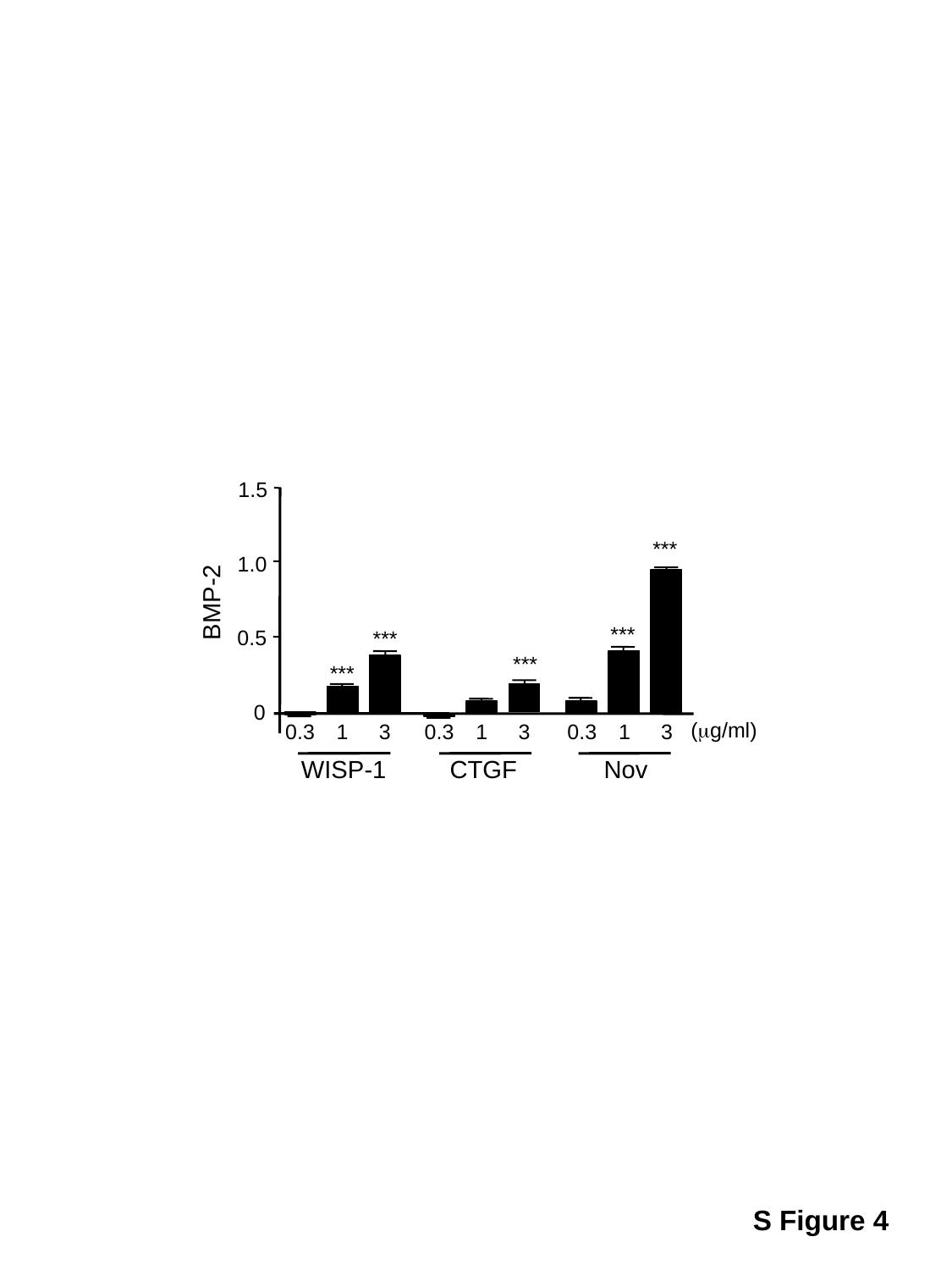

1.5
***
1.0
BMP-2
***
0.5
***
***
***
0
(μg/ml)
0.3
1
3
0.3
1
3
0.3
1
3
WISP-1
CTGF
Nov
S Figure 4

Supplement: Supplementary file 5 [file jbmr0026-0193-SD5.ppt]
