# Supplementary material for: WISP-1/CCN4 Regulates Osteogenesis by Enhancing BMP-2 Activity
Source: J Bone Miner Res. 2010 Aug 3;26(1):193–208. doi: 10.1002/jbmr.205 (PMC3179320; doi:10.1002/jbmr.205)
Supplement: Supplementary file 6 [file jbmr0026-0193-SD6.ppt]

## Slide 1
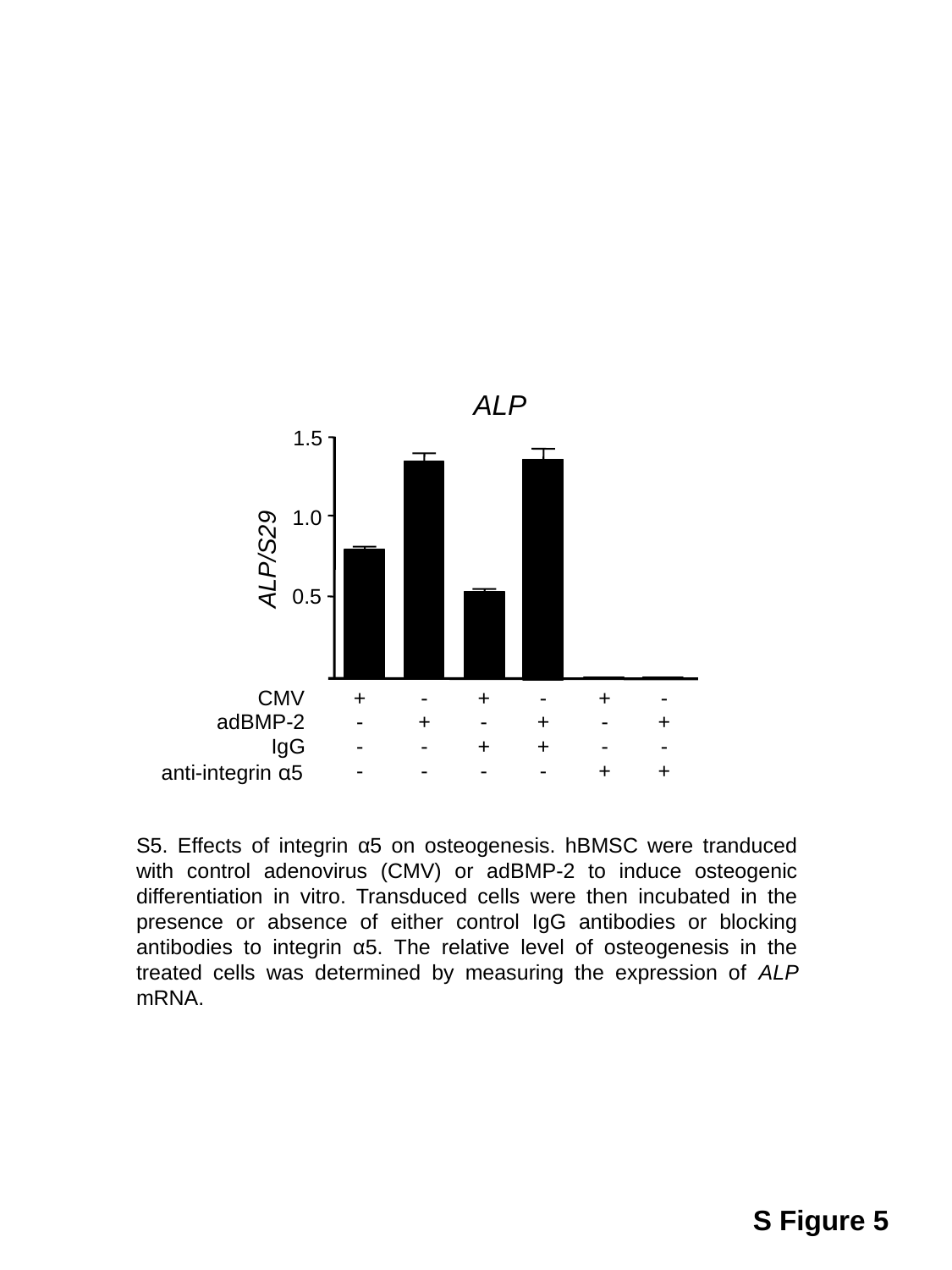

ALP
1.5
1.0
ALP/S29
0.5
CMV
+
-
+
-
+
-
adBMP-2
-
+
-
+
-
+
IgG
-
-
+
+
-
-
-
-
-
-
+
+
anti-integrin α5
S5. Effects of integrin α5 on osteogenesis. hBMSC were tranduced with control adenovirus (CMV) or adBMP-2 to induce osteogenic differentiation in vitro. Transduced cells were then incubated in the presence or absence of either control IgG antibodies or blocking antibodies to integrin α5. The relative level of osteogenesis in the treated cells was determined by measuring the expression of ALP mRNA.
S Figure 5
