# Supplementary material for: WISP-1/CCN4 Regulates Osteogenesis by Enhancing BMP-2 Activity
Source: J Bone Miner Res. 2010 Aug 3;26(1):193–208. doi: 10.1002/jbmr.205 (PMC3179320; doi:10.1002/jbmr.205)
Supplement: Supplementary file 7 [file jbmr0026-0193-SD7.ppt]

## Slide 1
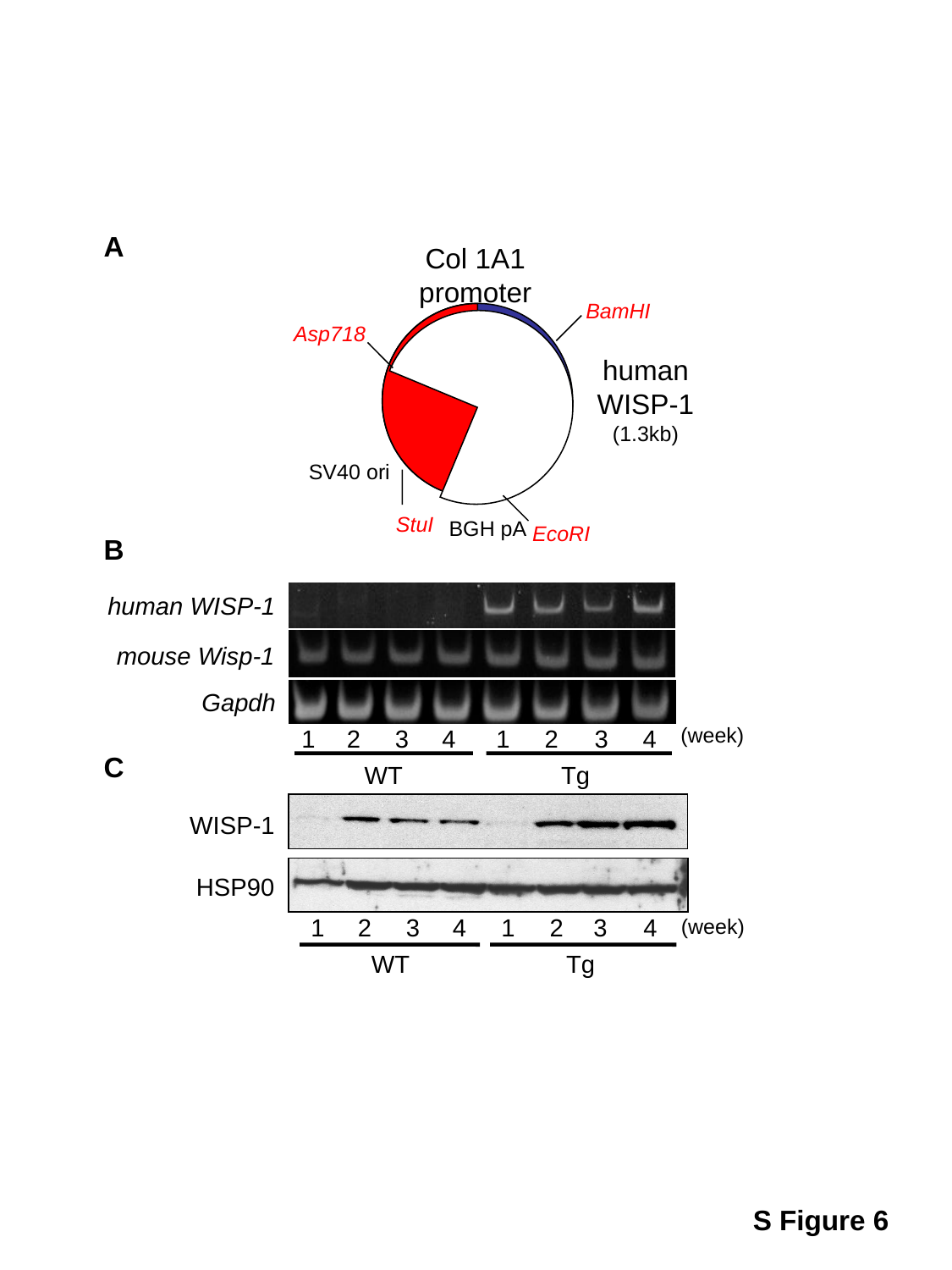

A
Col 1A1 promoter
 (2.4kb)
BamHI
Asp718
 human
WISP-1
(1.3kb)
pcDNA3.1+
SV40 ori
StuI
BGH pA
EcoRI
B
human WISP-1
mouse Wisp-1
Gapdh
(week)
1
2
3
4
1
2
3
4
C
WT
Tg
WISP-1
HSP90
1
2
3
4
1
2
3
4
(week)
WT
Tg
S Figure 6

## Slide 2
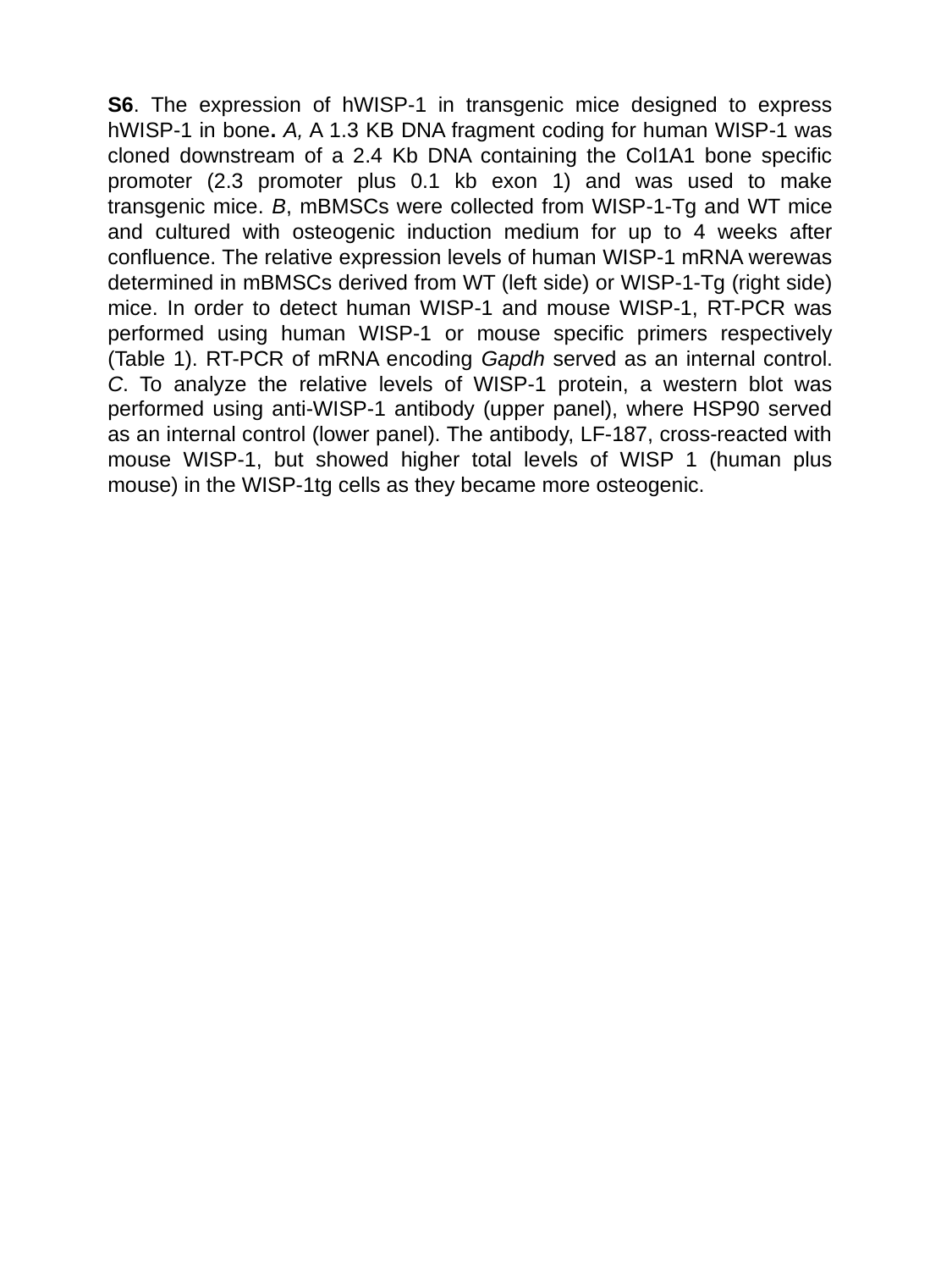

S6. The expression of hWISP-1 in transgenic mice designed to express hWISP-1 in bone. A, A 1.3 KB DNA fragment coding for human WISP-1 was cloned downstream of a 2.4 Kb DNA containing the Col1A1 bone specific promoter (2.3 promoter plus 0.1 kb exon 1) and was used to make transgenic mice. B, mBMSCs were collected from WISP-1-Tg and WT mice and cultured with osteogenic induction medium for up to 4 weeks after confluence. The relative expression levels of human WISP-1 mRNA werewas determined in mBMSCs derived from WT (left side) or WISP-1-Tg (right side) mice. In order to detect human WISP-1 and mouse WISP-1, RT-PCR was performed using human WISP-1 or mouse specific primers respectively (Table 1). RT-PCR of mRNA encoding Gapdh served as an internal control. C. To analyze the relative levels of WISP-1 protein, a western blot was performed using anti-WISP-1 antibody (upper panel), where HSP90 served as an internal control (lower panel). The antibody, LF-187, cross-reacted with mouse WISP-1, but showed higher total levels of WISP 1 (human plus mouse) in the WISP-1tg cells as they became more osteogenic.
