# Supplementary material for: WISP-1/CCN4 Regulates Osteogenesis by Enhancing BMP-2 Activity
Source: J Bone Miner Res. 2010 Aug 3;26(1):193–208. doi: 10.1002/jbmr.205 (PMC3179320; doi:10.1002/jbmr.205)
Supplement: Supplementary file 8 [file jbmr0026-0193-SD8.ppt]

## Slide 1
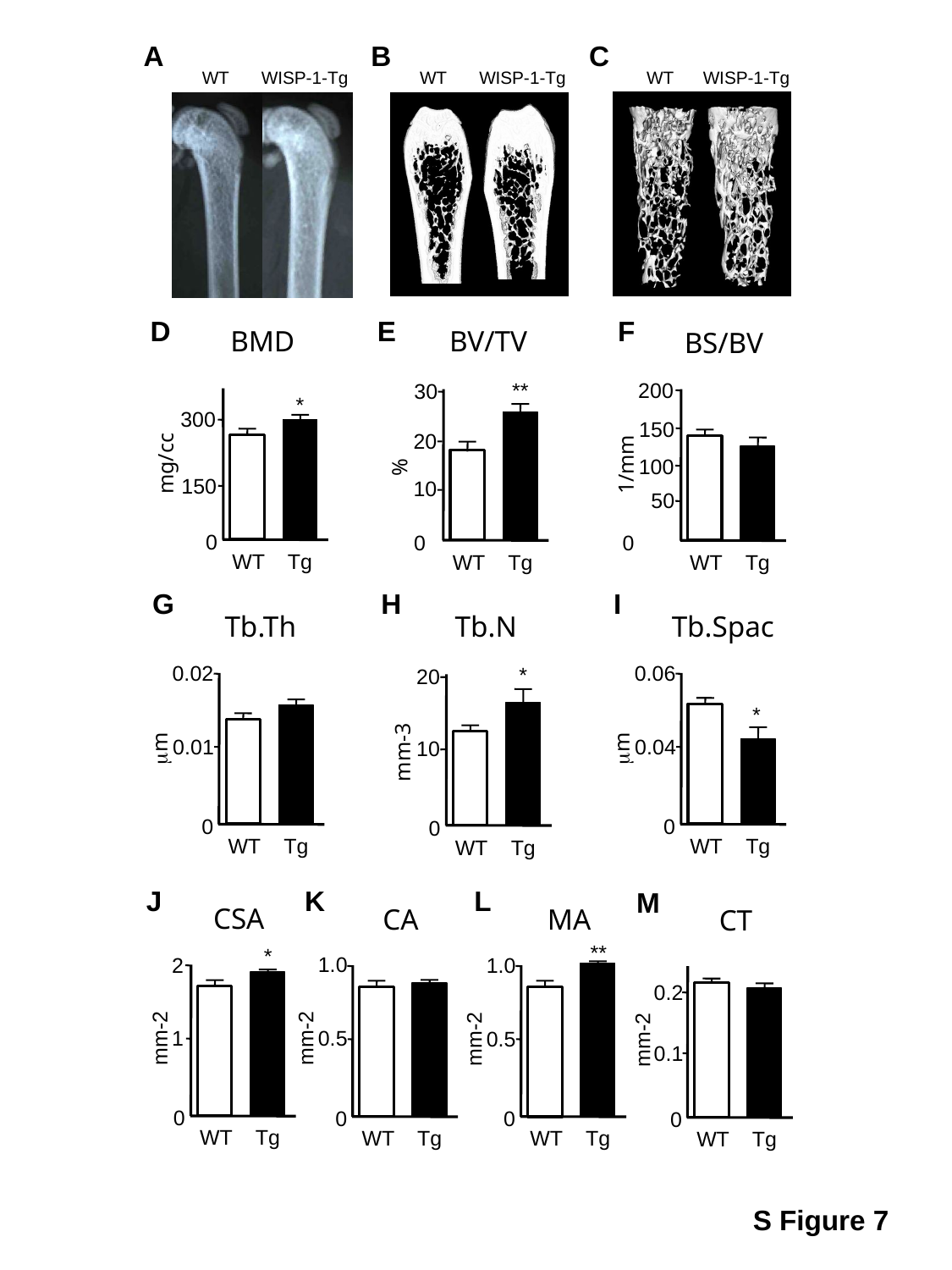

A
B
C
WT
WISP-1-Tg
WT
WISP-1-Tg
WT
WISP-1-Tg
D
E
F
BMD
BV/TV
BS/BV
**
200
30
*
300
150
20
mg/cc
1/mm
100
%
150
10
50
0
0
0
WT
Tg
WT
Tg
WT
Tg
G
H
I
Tb.Th
Tb.N
Tb.Spac
0.02
0.06
*
20
*
0.01
0.04
10
μm
μm
mm-3
0
0
0
WT
Tg
WT
Tg
WT
Tg
J
K
L
M
CSA
CA
MA
CT
**
*
1.0
2
1.0
0.2
0.5
1
mm-2
mm-2
0.5
mm-2
mm-2
0.1
0
0
0
0
WT
Tg
WT
Tg
WT
Tg
WT
Tg
S Figure 7

## Slide 2
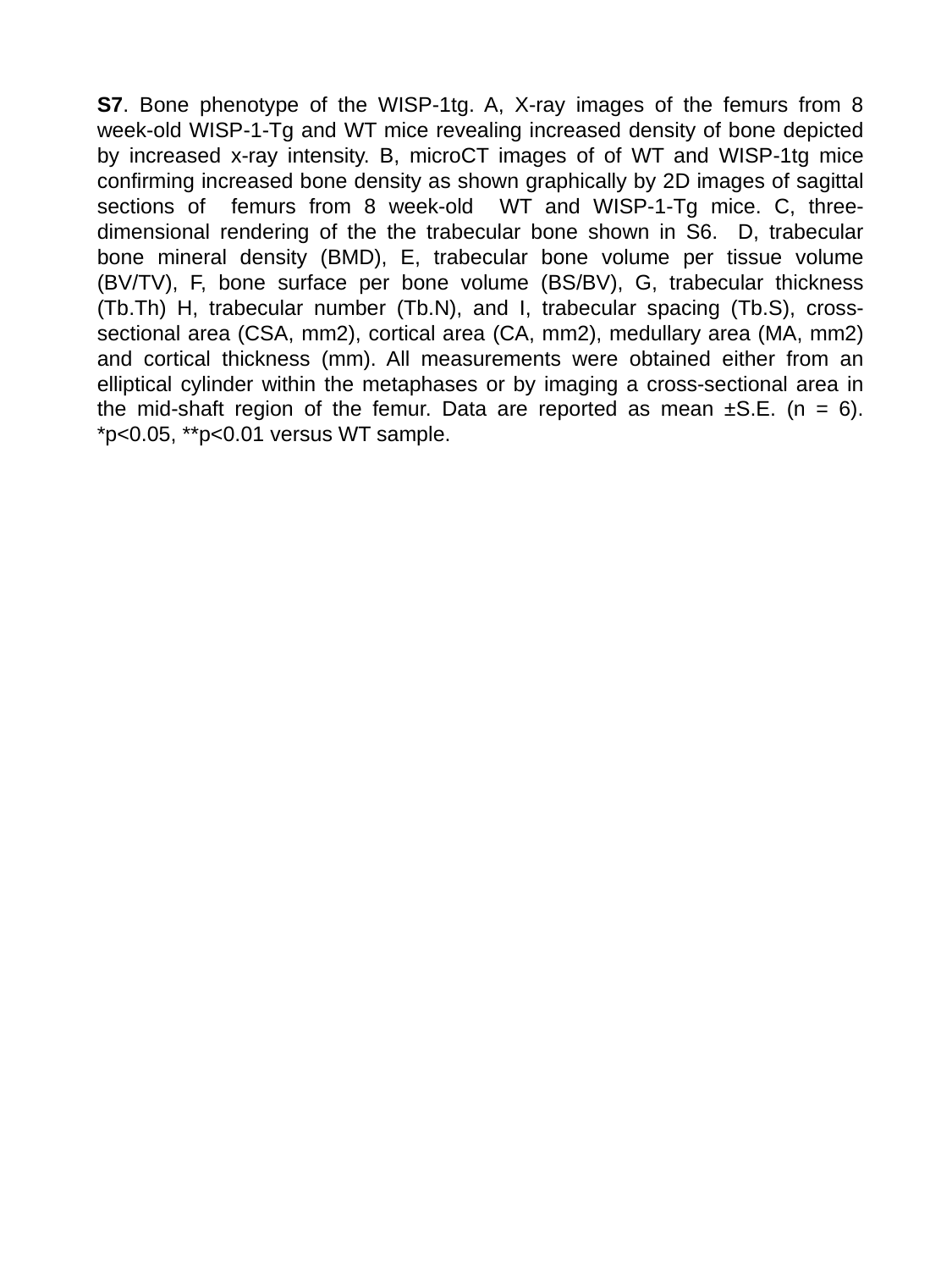

S7. Bone phenotype of the WISP-1tg. A, X-ray images of the femurs from 8 week-old WISP-1-Tg and WT mice revealing increased density of bone depicted by increased x-ray intensity. B, microCT images of of WT and WISP-1tg mice confirming increased bone density as shown graphically by 2D images of sagittal sections of femurs from 8 week-old WT and WISP-1-Tg mice. C, three-dimensional rendering of the the trabecular bone shown in S6. D, trabecular bone mineral density (BMD), E, trabecular bone volume per tissue volume (BV/TV), F, bone surface per bone volume (BS/BV), G, trabecular thickness (Tb.Th) H, trabecular number (Tb.N), and I, trabecular spacing (Tb.S), cross-sectional area (CSA, mm2), cortical area (CA, mm2), medullary area (MA, mm2) and cortical thickness (mm). All measurements were obtained either from an elliptical cylinder within the metaphases or by imaging a cross-sectional area in the mid-shaft region of the femur. Data are reported as mean ±S.E. (n = 6). *p<0.05, **p<0.01 versus WT sample.
